# 940-nm Photobiomodulation Enhances ADSC-Mediated Tracheal Cartilage Repair through TRPV1-Associated Calcium Signaling and Mitochondrial Redox Remodeling

**DOI:** 10.34133/research.1440

**Published:** 2026-10-06

**Authors:** Yong Xu, Erji Gao, Zhe-Sheng Chen, Bo Tao

**Affiliations:** ^1^ Department of Thoracic Surgery, Shanghai Pulmonary Hospital, Tongji University School of Medicine, Shanghai 200443, China.; ^2^ Department of Pharmaceutical Science, College of Pharmacy and Health Science, St John’s University, Queens, NY 11439, USA.

## Abstract

Tracheal cartilage repair requires simultaneous restoration of a stable cartilage matrix, airway patency, and mechanical support, yet the regenerative state of implanted stem cells remains difficult to control. Here, we show that 940-nm photobiomodulation (PBM) enhances the chondrogenic competence of adipose-derived stem cells (ADSCs) within a defined dose window. Among the radiant exposures tested, 50 J/cm^2^ most effectively promoted cell-cycle progression, cartilage-matrix formation and expression of SRY-box transcription factor 9, type II collagen, and aggrecan, while suppressing fibrotic, hypertrophic, and catabolic markers. These effects were not reproduced by a temperature-matched heat control. In 3-dimensional porous scaffolds, preconditioned ADSCs exhibited improved survival, spreading, matrix deposition, and mechanical performance. Mechanistically, 940-nm PBM remodeled mitochondrial architecture, membrane potential, respiration, cellular adenosine triphosphate content and redox status. Pathway perturbation implicated transient receptor potential vanilloid 1-associated Ca^2+^ signaling, a redox-dependent component of Akt/glycogen synthase kinase 3β activation and SRY-box transcription factor 9 nuclear localization in the chondrogenic response. Preconditioning also preserved cartilage-associated gene expression under inflammatory challenge and altered macrophage-associated inflammatory marker profiles. In a rabbit tracheal cartilage defect model, constructs preconditioned at 50 J/cm^2^ improved cartilage-like tissue formation, epithelial repopulation, lumen patency, and mechanical function. These findings support repeated 940-nm PBM conditioning as a controllable ex vivo strategy for enhancing ADSC chondrogenic competence and improving early tracheal cartilage repair across cellular and tissue scales.

## Introduction

Segmental loss or structural disruption of tracheal cartilage can compromise airway patency and expose the respiratory tract to progressive stenosis, dynamic collapse, and recurrent obstruction [1]. Unlike many cartilaginous tissues, the trachea must simultaneously maintain a mechanically stable lumen, accommodate repetitive deformation during respiration, and support restoration of a functional airway epithelium [2]. These requirements make tracheal cartilage regeneration particularly demanding: Successful repair cannot be defined solely by extracellular matrix (ECM) deposition but must also involve restoration of tissue architecture, resistance to collapse, and integration with the surrounding airway wall [3]. Current tissue-engineering strategies commonly combine mesenchymal stromal cells with porous scaffolds or hydrogels to reconstruct damaged cartilage [4]. However, the therapeutic performance of these constructs is often limited by the functional state of the implanted cells [5]. Cells delivered into a defect may remain incompletely differentiated, undergo fibrotic or hypertrophic conversion, or lose their regenerative phenotype in response to inflammatory and mechanical stress [6,7]. Thus, a central challenge is not simply to deliver more cells but to establish a cell state that is competent to generate stable cartilage-like tissue under the adverse conditions encountered after implantation.

Adipose-derived stem cells (ADSCs) are attractive for cartilage regeneration because they are abundant, readily accessible, and capable of chondrogenic differentiation [8]. Nevertheless, ADSC chondrogenesis is not intrinsically stable. Although biochemical induction can increase the expression of cartilage-associated molecules such as SRY-box transcription factor 9 (SOX9), type II collagen (COL II), and aggrecan, differentiation may be accompanied by type I collagen (COL I)-rich fibrotic matrix formation, type X collagen (COL X) and runt-related transcription factor 2 (RUNX2)-associated hypertrophy, and matrix metallopeptidase 13 (MMP13)-mediated matrix degradation [9,10]. These maladaptive programs are particularly problematic for tracheal reconstruction, where mechanically weak or phenotypically unstable tissue is unlikely to provide durable airway support. Transforming growth factor-β3 (TGF-β3) is widely used to promote mesenchymal stem-cell chondrogenesis, but soluble growth-factor stimulation offers limited temporal and spatial control and does not directly address the metabolic and redox conditions required to sustain matrix synthesis [11]. A controllable physical preconditioning strategy that modulates cell state without requiring the addition of a soluble agonist during the stimulation step could therefore complement conventional biochemical approaches for improving the functional quality of ADSC-based constructs.

Photobiomodulation (PBM) uses low-intensity visible or near-infrared light to regulate cellular behavior without causing overt photothermal injury [12]. Because light can be delivered in a spatially confined, temporally controlled, and readily withdrawn manner, PBM provides a physical means of modulating cell function during ex vivo culture. Previous studies have reported that PBM can influence stem-cell proliferation, mitochondrial activity, oxidative stress, and lineage differentiation [13]. However, much of the field remains descriptive, and the cellular mechanisms that convert a defined optical input into a lineage-specific regenerative response are incompletely understood. Biological effects vary substantially with wavelength, radiant exposure, irradiation schedule, and cellular context, indicating that PBM does not act as a uniform metabolic stimulant [14]. The prevailing mitochondrial model proposes that light-responsive respiratory-chain components, including cytochrome-c oxidase (CCO), may contribute to changes in mitochondrial respiration, adenosine triphosphate (ATP) generation, and reactive oxygen species (ROS) signaling [15]. Yet, this model does not fully explain the wavelength- and cell-type-dependent nature of PBM, and alternative membrane-associated mechanisms have increasingly been implicated.

Transient receptor potential vanilloid 1 (TRPV1) is a Ca^2+^-permeable membrane channel capable of coupling extracellular physical cues to intracellular calcium signaling [16]. Ca^2+^ influx can rapidly influence mitochondrial membrane potential, respiratory activity, cellular ATP content, and redox homeostasis, thereby linking membrane excitation to metabolic regulation [17]. These mitochondrial changes may, in turn, engage signaling pathways that control cell fate. In cartilage differentiation, protein kinase B (Akt) activation and inhibitory Ser9 phosphorylation of glycogen synthase kinase 3β (GSK3β) can support SOX9 stabilization and nuclear activity, promoting cartilage-matrix gene expression [18]. This raises the possibility that near-infrared light does not merely increase cellular metabolism but coordinates a membrane-to-mitochondria signaling response that establishes a metabolically permissive condition for chondrogenesis. Importantly, such a mechanism would be expected to operate within a restricted dose window. Insufficient stimulation may fail to activate the required bioenergetic response, whereas excessive irradiation may disturb redox balance and diminish regenerative performance.

Here, we investigated whether 940-nm PBM could improve the regenerative competence of ADSCs for functional tracheal cartilage repair. We first established a calibrated irradiation platform and compared radiant exposures of 0, 25, 50, and 100 J/cm^2^, using TGF-β3 as a chondrogenic reference and a temperature-matched heat-control condition to distinguish photobiomodulatory effects from nonspecific thermal responses. We found that an intermediate radiant exposure of 50 J/cm^2^ provided the most favorable biological window, enhancing ADSC proliferation, cartilage-associated matrix production, and SOX9 activity while limiting fibrotic, hypertrophic, and catabolic phenotypes. When transferred to egg white-derived porous scaffolds, PBM-conditioned ADSCs exhibited improved survival, spreading, cartilage-like matrix formation, and mechanical performance. Mechanistically, 940-nm PBM altered mitochondrial architecture, membrane potential, respiratory activity, cellular ATP content, and ROS signaling. Pharmacological and genetic perturbation further implicated TRPV1-associated Ca^2+^ signaling, redox regulation, and the Akt/GSK3β–SOX9 axis in the chondrogenic response. 940-nm PBM also preserved cartilage-associated gene expression under interleukin-1 beta (IL-1β) challenge and modulated inflammatory macrophage phenotypes. Finally, implantation of preconditioned ADSC–scaffold constructs improved cartilage-like tissue formation, epithelial repopulation and differentiation, lumen patency, and mechanical function in a tracheal cartilage defect model (Fig. 1). The principal objective of this study was therefore not simply to determine whether 940-nm PBM promotes chondrogenesis but to test whether a defined optical intervention could be used to establish a more regenerative ADSC state that remains functionally relevant when transferred from cell culture to an engineered construct and ultimately to tracheal repair. The dose-response, TRPV1-associated Ca^2+^ signaling, and mitochondrial-redox analyses were used to characterize and mechanistically contextualize this PBM-associated cellular response.

**Fig. 1.**
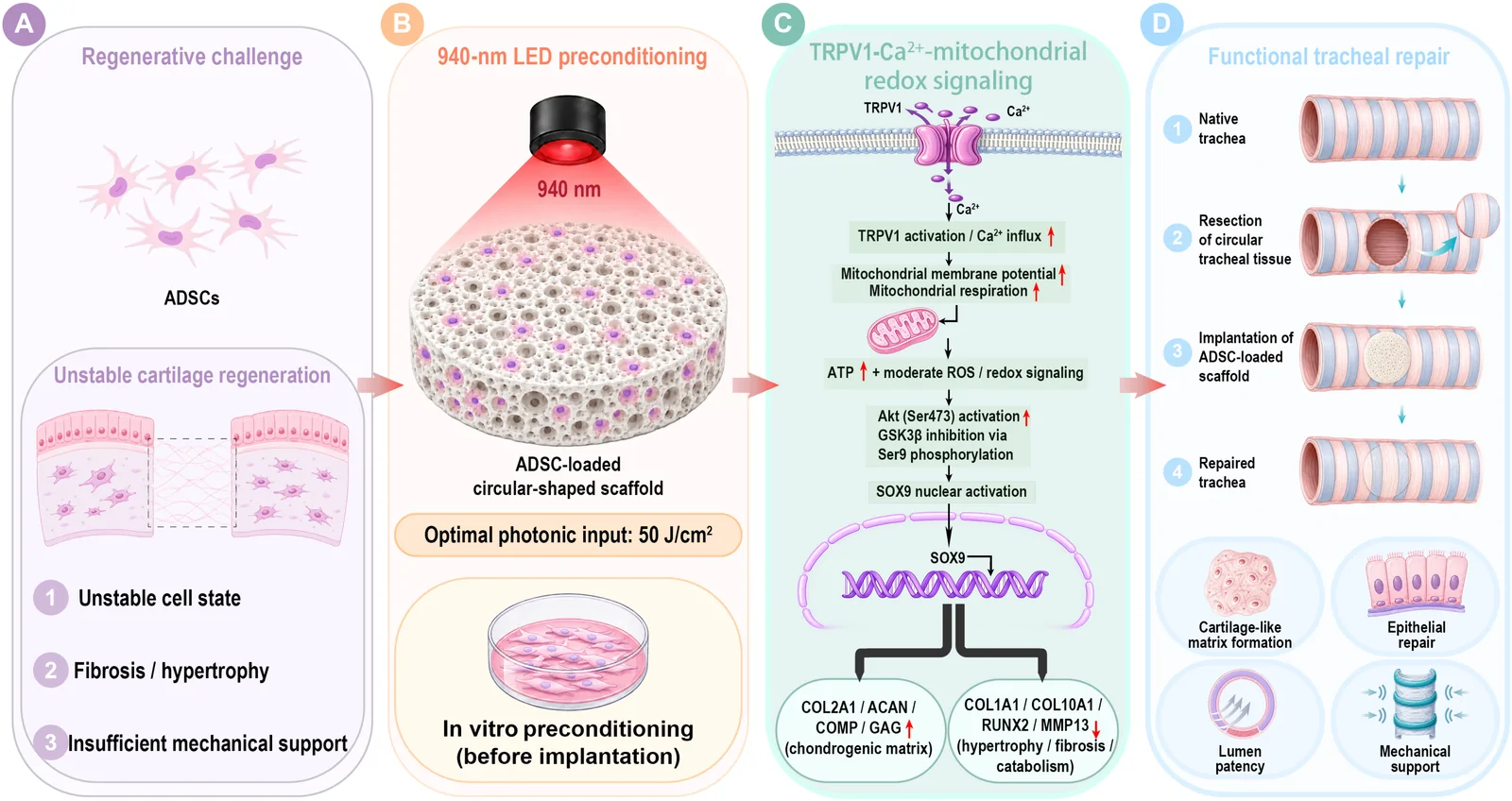
Working model illustrating how repeated 940-nm photobiomodulation (PBM) conditioning enhances adipose-derived stem cell (ADSC)–scaffold construct remodeling and tracheal cartilage repair. (A) Schematic overview of the challenges associated with ADSC-based tracheal cartilage repair, including limited chondrogenic commitment, insufficient extracellular matrix formation, and inadequate mechanical restoration. (B) Repeated 940-nm PBM treatment during culture enhances ADSC regenerative competence within the egg white-derived porous scaffold microenvironment. The scaffold provides structural support and a permissive 3-dimensional niche for cell attachment, migration, matrix deposition, and subsequent tissue remodeling. (C) Working model illustrating the proposed cellular mechanisms underlying 940-nm PBM. Irradiation engages transient receptor potential vanilloid 1 (TRPV1)-associated calcium signaling, mitochondrial bioenergetic/redox remodeling, and Akt/glycogen synthase kinase 3β (GSK3β)–SRY-box transcription factor 9 (SOX9) regulation, which collectively contribute to enhanced chondrogenic phenotype acquisition. (D) Implantation of irradiation-conditioned ADSC–scaffold constructs promotes early tracheal cartilage repair, including cartilage-like extracellular matrix formation, epithelial repopulation, airway lumen preservation, and improved mechanical support. The observed tissue-level outcomes represent the combined contribution of scaffold-mediated structural support, irradiation-modulated cellular activity, and progressive scaffold–tissue remodeling.

## Results and Discussion

### A calibrated 940-nm irradiation window enhances ADSC proliferative competence

Before investigating the effects of 940-nm PBM, we verified the identity and differentiation potential of the ADSCs used in this study. Passage-3 cells displayed the expected spindle-shaped morphology (Fig. S1A and B), expressed the mesenchymal markers CD29, CD44, CD73, and CD105, showed low expression of CD34 and human leukocyte antigen-DR (Fig. S1C and, D), and retained adipogenic, osteogenic, and chondrogenic differentiation capacity (Fig. S1E and F). These findings confirmed that the cells possessed the phenotypic characteristics required for subsequent PBM and cartilage-engineering experiments.

We next established a standardized light-emitting diode (LED) irradiation platform that enabled precise control and monitoring of the source-to-sample distance, output power, and sample temperature (Fig. 2A). ADSCs were assigned to the 0, 25, 50, and 100 J/cm^2^ irradiation groups, a TGF-β3 chondrogenic-reference group, and a heat-control (HC) group. The 0 J/cm^2^ group underwent the same experimental handling without light exposure, whereas the TGF-β3 group served as a reference for chondrogenic induction [19]. To distinguish photobiomodulatory effects from nonspecific thermal effects, the HC group was not irradiated but was exposed to a temperature–time profile matched to that measured in the 50 J/cm^2^ group. Culture-medium temperature increased progressively with radiant exposure, with the greatest increase observed in the 100 J/cm^2^ group (Fig. S2A and B). Importantly, the HC group closely reproduced the temperature profile of the 50 J/cm^2^ condition in the absence of light, thereby allowing the biological effects of the best-performing tested condition to be separated from those attributable to mild heating alone.

**Fig. 2.**
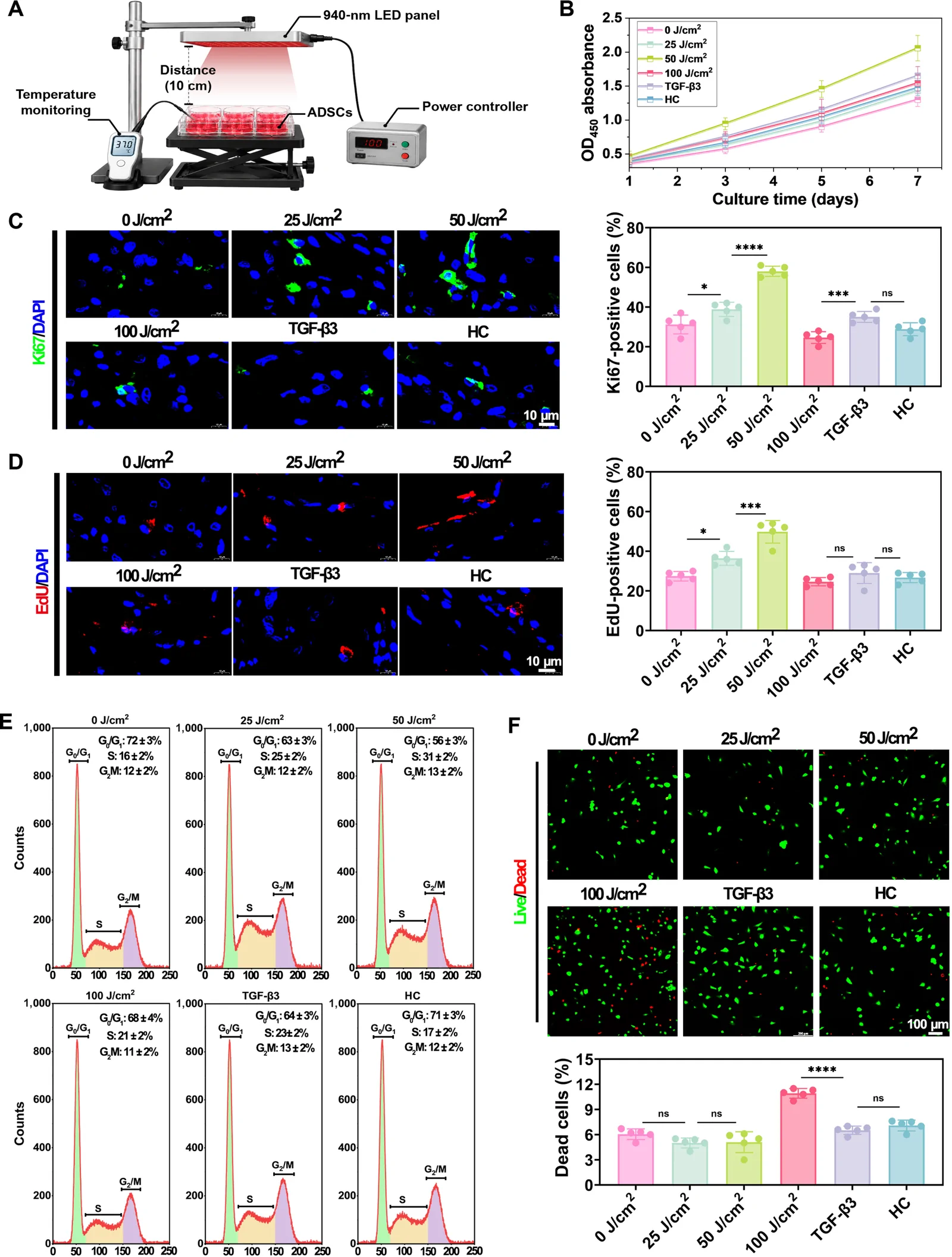
Photobiomodulation (PBM) at 940 nm regulates adipose-derived stem cell (ADSC) proliferation, cell-cycle progression, and viability in a dose-dependent manner. (A) Schematic of the 940-nm PBM platform, including a fixed 10-cm source-to-sample distance, a power controller and real-time temperature monitoring. (B) Cell Counting Kit-8 (CCK-8) growth curves of ADSCs exposed to 0, 25, 50, or 100 J/cm^2^, transforming growth factor-β3 (TGF-β3) or heat control (HC). (C) Representative Ki67 immunofluorescence images and quantification of Ki67-positive cells. Ki67, green; 4′,6-diamidino-2-phenylindole (DAPI), blue. (D) Representative 5-ethynyl-2′-deoxyuridine (EdU) incorporation images and quantification of EdU-positive cells. EdU, red; DAPI, blue. (E) Flow-cytometric cell-cycle analysis showing the proportions of cells in the G_0_/G_1_, S, and G_2_/M phases. (F) Representative Live/Dead staining images and quantification of dead cells. Live cells, green; dead cells, red. Data in (B) to (D) and (F) are presented as mean ± SD; *n* = 5 independent culture-level experimental units derived from the same ADSC preparation. Time-course data in (B) were analyzed by 2-way analysis of variance (ANOVA), whereas endpoint data in (C), (D), and (F) were analyzed by 1-way ANOVA followed by Tukey’s multiple-comparisons test. Horizontal brackets indicate the comparisons tested. ns, not significant; *P < 0.05; ***P < 0.001; ****P < 0.0001.

ADSC proliferation exhibited a nonlinear response to 940-nm irradiation. Cell Counting Kit-8 (CCK-8) measurements showed that 50 J/cm^2^ produced the most pronounced increase in cell growth over the culture period, whereas 25 J/cm^2^ induced a more modest response and 100 J/cm^2^ failed to provide a comparable benefit (Fig. 2B). Consistent with these findings, the proportions of Ki67-positive and 5-ethynyl-2′-deoxyuridine (EdU)-positive cells were highest after 50 J/cm^2^ irradiation (Fig. 2C and D). Flow-cytometric analysis further showed an increased proportion of cells in S phase under this condition, indicating enhanced progression through the G_1_/S transition rather than a nonspecific increase in metabolic assay signals (Fig. 2E and Fig. S3A and B).

The enhanced proliferative response at 50 J/cm^2^ was not reproduced by the temperature-matched HC condition. This observation argues against mild thermal elevation as the principal explanation for the increased cell-cycle activity. At the same time, live/dead staining showed that most cells remained viable across the tested conditions, although the 100 J/cm^2^ group contained a higher proportion of dead cells than the lower-dose groups (Fig. 2F). Thus, the reduced biological benefit observed at 100 J/cm^2^ likely reflects departure from the best-performing tested condition and may involve both excessive photobiological stimulation and the greater thermal burden associated with prolonged irradiation.

Together, these results reveal a dose-restricted response in which an intermediate radiant exposure promotes ADSC proliferation and cell-cycle progression, whereas a higher dose provides no additional benefit and begins to compromise cellular fitness. Such a pattern is consistent with the biphasic behavior reported for PBM in other cell systems [20]. More importantly, it establishes that radiant exposure is not merely a technical variable in this study, but a determinant of the cellular state subsequently used for cartilage formation. On the basis of this initial screening, 50 J/cm^2^ was selected as the principal preconditioning dose for mechanistic and regenerative evaluation.

The observed dose window should be interpreted within the specific irradiation protocol used here. Because irradiance was maintained at 100 mW/cm^2^, increasing radiant exposure from 25 to 50 and 100 J/cm^2^ necessarily increased exposure duration from 250 to 500 and 1,000 s, respectively; thus, radiant exposure and irradiation time were experimentally coupled. Repeated daily treatment also introduced cumulative-exposure effects, while longer irradiation was accompanied by greater temperature elevation. The absence of a comparable response in the HC condition matched to 50 J/cm^2^ argues against mild heating as the principal explanation for the favorable response at this condition; however, a contribution of the greater thermal burden at 100 J/cm^2^ cannot be excluded. Accordingly, 50 J/cm^2^ should be regarded as the best-performing operational condition among those tested rather than as a universal or precisely defined optimum. Intermediate radiant exposures, including conditions between 50 and 100 J/cm^2^, were not examined and may further refine the treatment window.

### Intermediate-dose irradiation promotes matrix-rich ADSC chondrogenesis

We next asked whether the proliferative advantage induced by 940-nm PBM was accompanied by productive chondrogenic differentiation. ADSCs were assembled into 3-dimensional (3D) pellets, exposed to the indicated irradiation conditions once daily and maintained for 14 d, with TGF-β3 serving as a chondrogenic reference condition (Fig. 3A).

**Fig. 3.**
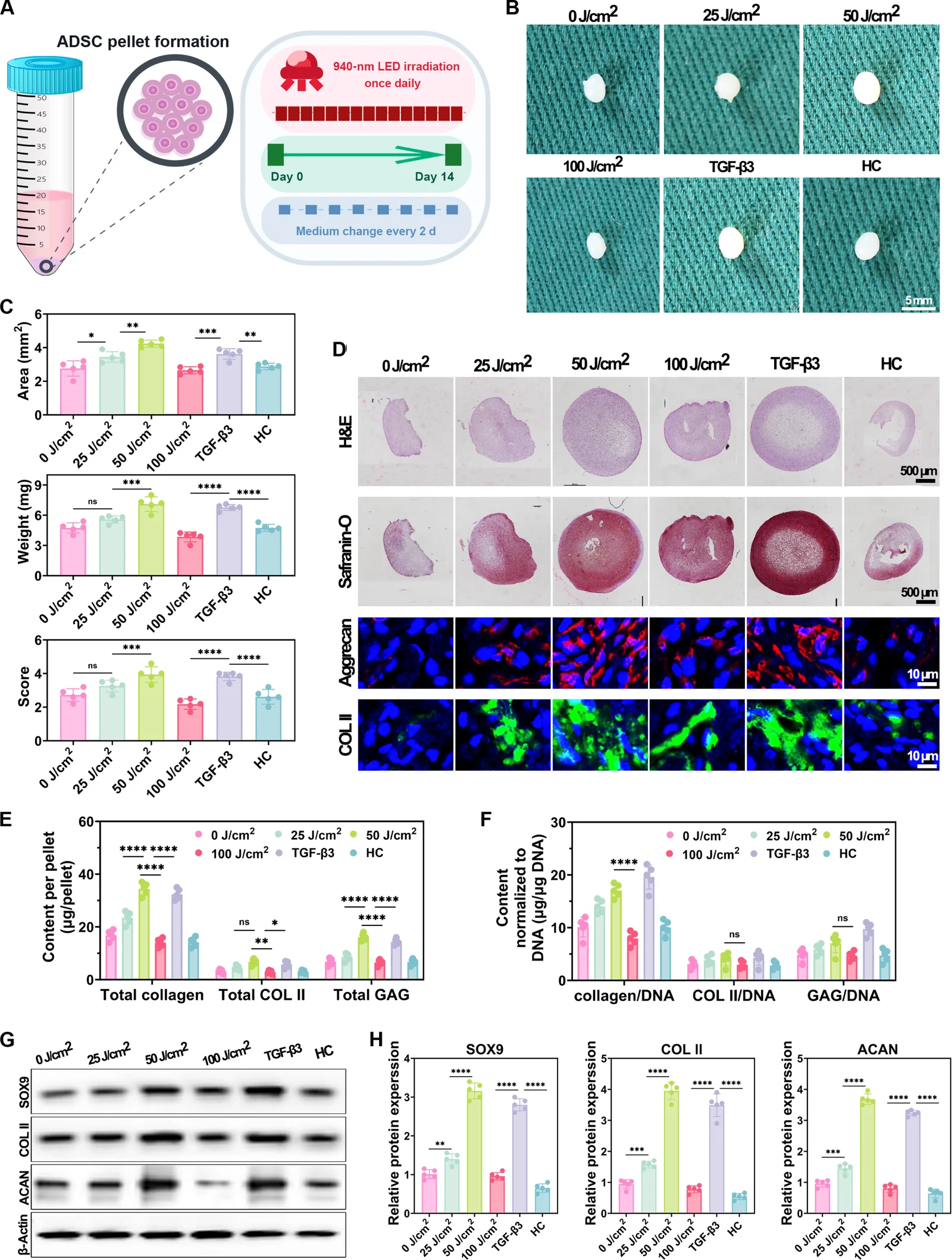
Photobiomodulation (PBM) at 940 nm enhances chondrogenic differentiation and cartilage-matrix production in adipose-derived stem cell (ADSC) pellets. (A) Experimental scheme for ADSC pellet formation, once-daily 940-nm PBM and 14 d of culture, with medium replacement every 2 d. (B) Gross appearance of pellets treated with 0, 25, 50, or 100 J/cm^2^, transforming growth factor-β3 (TGF-β3) or heat control (HC). (C) Quantification of pellet area, wet weight, and macroscopic compactness score. (D) Representative hematoxylin and eosin (H&E) and Safranin O staining, together with aggrecan and type II collagen (COL II) immunofluorescence. Nuclei were counterstained with 4′,6-diamidino-2-phenylindole (DAPI). (E) Total collagen, COL II, and glycosaminoglycan (GAG) content per pellet. (F) Collagen, COL II, and GAG content normalized to DNA. (G) Immunoblot analysis of SRY-box transcription factor 9 (SOX9), COL II, and ACAN, with β-actin as the loading control. (H) Densitometric quantification of the indicated proteins. For continuous outcomes, data are presented as mean ± SD; macroscopic compactness scores are presented as median with interquartile range. *n* = 5 independent pellets derived from the same ADSC preparation. Continuous outcomes were analyzed by 1-way analysis of variance (ANOVA) followed by Tukey’s multiple-comparisons test, whereas macroscopic compactness scores were analyzed using the Kruskal–Wallis test followed by Dunn’s multiple-comparisons test. Horizontal brackets indicate the comparisons tested. ns, not significant; *P < 0.05; **P < 0.01; ***P < 0.001; ****P < 0.0001.

Gross examination showed that pellets exposed to 50 J/cm^2^ were larger and comparable in size to those in the TGF-β3 group (Fig. 3B). Quantitative analysis further demonstrated that the 50 J/cm^2^ group had a greater surface area, higher wet weight, and a more compact macroscopic appearance than the untreated, 100 J/cm^2^, and HC groups (Fig. 3C). Although pellet enlargement may result from increased cell number, ECM accumulation, or both, the histological and biochemical findings indicated that the response extended beyond simple expansion of the cell population. Hematoxylin and eosin (H&E) staining revealed a denser and more coherent tissue architecture, whereas Safranin O staining showed pronounced proteoglycan deposition in the 50 J/cm^2^ group (Fig. 3D and Fig. S4). Notably, although the overall chondrogenic responses of the 50 J/cm^2^ and TGF-β3 groups were comparable, matrix deposition appeared more spatially homogeneous in the 50 J/cm^2^ group. By contrast, the central region of some TGF-β3-treated pellets showed relatively weak cartilage-matrix staining, potentially reflecting diffusion-related limitations in nutrient or growth-factor availability within the pellet core. This observation suggests that the 2 interventions may differ in how they regulate matrix formation within the pellet microenvironment, although the basis of this spatial difference was not resolved. Aggrecan and COL II immunofluorescence were also increased, supporting the formation of cartilage-associated ECM rather than nonspecific connective tissue.

Biochemical measurements confirmed that total collagen, COL II, and glycosaminoglycan (GAG) contents were increased after 50 J/cm^2^ irradiation (Fig. 3E). Crucially, these differences remained evident after normalization to DNA content (Fig. 3F). The increased collagen/DNA, COL II/DNA, and GAG/DNA ratios indicate that the enhanced matrix content could not be explained solely by the greater number of proliferating cells. Instead, irradiation increased the matrix-producing activity of individual cells or promoted their entry into a more productive chondrogenic state.

At the molecular level, SOX9, COL II, and ACAN protein abundance was highest or among the highest in the 50 J/cm^2^ group (Fig. 3G and H). SOX9 is a central transcriptional regulator of the chondrocyte lineage, whereas COL II and ACAN are principal structural components of cartilage matrix [21]. Their coordinated induction therefore supports activation of a coherent chondrogenic program rather than isolated changes in matrix composition.

The comparison with TGF-β3 is informative. TGF-β3 produced the expected chondrogenic response, validating the sensitivity of the pellet model. However, the 50 J/cm^2^ group achieved a strong cartilage-associated phenotype without the pattern being reproduced by the HC group. These data suggest that 940-nm irradiation acts through a light-dependent biological process and can complement the type of lineage regulation conventionally achieved using soluble induction factors.

An important implication is that the best-performing tested condition for chondrogenesis was not defined merely by pellet size or bulk collagen deposition. The persistence of the effect after normalization to DNA, together with the coordinated increase in SOX9, COL II, ACAN, and GAG, indicates that 50 J/cm^2^ improved the quality of the cartilage-forming response. This distinction is particularly relevant to tissue engineering, because an enlarged construct that contains poorly organized or noncartilaginous matrix would not necessarily provide functional mechanical support.

### Intermediate-dose PBM suppresses fibrotic, hypertrophic, and catabolic drift during chondrogenesis

Mesenchymal stromal-cell chondrogenesis is frequently accompanied by undesirable lineage drift [22]. Increased matrix production alone is therefore insufficient to establish phenotypic stability, particularly when fibrotic collagen, hypertrophic differentiation, and matrix-degrading enzymes are also induced [23]. We consequently examined whether 940-nm PBM altered these maladaptive programs.

Immunofluorescence analysis showed that collagen type I alpha 1 chain (COL1A1), collagen type X alpha 1 chain (COL10A1), RUNX2, and MMP13 signals were lower in the 50 J/cm^2^ group than in the untreated and 100 J/cm^2^ groups (Fig. 4A). Immunoblotting and densitometric analysis confirmed this pattern at the protein level (Fig. 4B and C), while reverse transcription quantitative polymerase chain reaction (RT-qPCR) demonstrated corresponding changes in *Col1a1*, *Col10a1*, *Runx2*, and *Mmp13* transcripts (Fig. 4D).

**Fig. 4.**
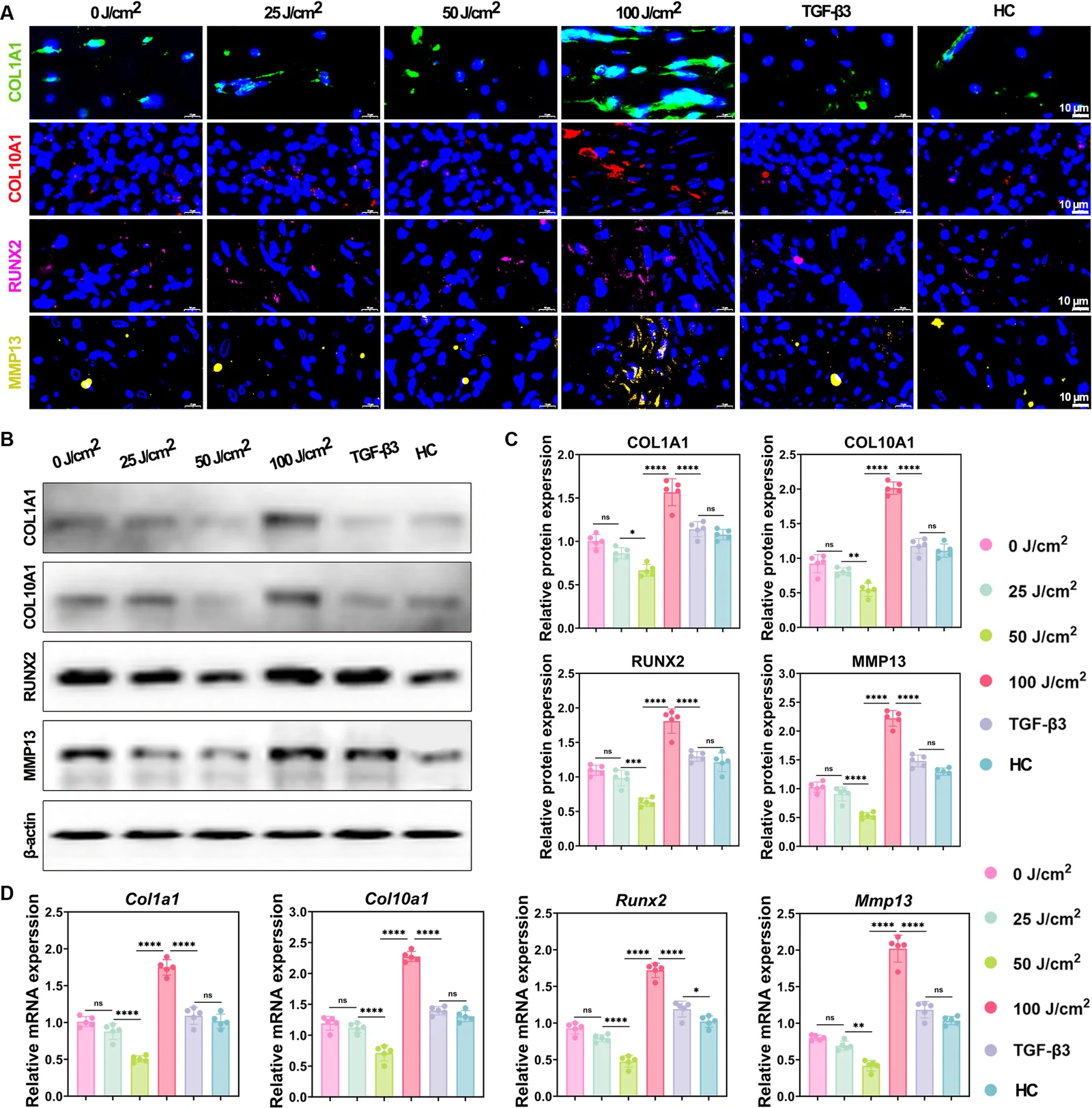
An intermediate 940-nm photobiomodulation (PBM) dose suppresses fibrotic, hypertrophic, and catabolic features during adipose-derived stem cell (ADSC) chondrogenesis. (A) Representative immunofluorescence images of collagen type I alpha 1 chain (COL1A1), collagen type X alpha 1 chain (COL10A1), runt-related transcription factor 2 (RUNX2), and matrix metallopeptidase 13 (MMP13) in ADSC pellets subjected to the indicated irradiation conditions, transforming growth factor-β3 (TGF-β3), or heat control (HC). Nuclei were counterstained with 4′,6-diamidino-2-phenylindole (DAPI). (B) Immunoblot analysis of COL1A1, COL10A1, RUNX2, and MMP13, with β-actin as the loading control. (C) Densitometric quantification of the proteins shown in (B). (D) reverse transcription quantitative polymerase chain reaction (RT-qPCR) analysis of *Col1a1*, *Col10a1*, *Runx2*, and *Mmp13* expression. Data are presented as mean ± SD; *n* = 5 independent culture-level experimental units derived from the same ADSC preparation. Statistical comparisons were performed by 1-way analysis of variance (ANOVA) followed by Tukey’s multiple-comparisons test. Horizontal brackets indicate the comparisons tested. ns, not significant; *P < 0.05; **P < 0.01; ***P < 0.001; ****P < 0.0001.

These markers represent distinct but interconnected threats to stable cartilage formation. COL1A1 is associated with fibrocartilaginous or fibrotic matrix, COL10A1 and RUNX2 indicate hypertrophic progression, and MMP13 promotes degradation of cartilage ECM [24]. Their simultaneous suppression suggests that 50 J/cm^2^ irradiation did not simply accelerate differentiation along an otherwise unchanged trajectory. Rather, it altered the balance between cartilage-specific matrix synthesis and competing fibrotic, hypertrophic, and catabolic programs.

To further evaluate the balance between cartilage-associated and fibrotic collagen programs, we calculated the relative *Col2a1/Col1a1* expression ratio from the corresponding RT-qPCR measurements. The ratio was markedly increased under the 50 J/cm^2^ condition and was substantially lower at 100 J/cm^2^ (Fig. S5), indicating a more favorable transcriptional balance toward type II rather than type I collagen at the intermediate exposure. TGF-β3 also produced a high *Col2a1/Col1a1* ratio, consistent with its role as a chondrogenic reference. This analysis complements the COL II/DNA measurements in Fig. 3F: COL II/DNA evaluates cartilage-matrix production relative to cell content, whereas the *Col2a1/Col1a1* ratio provides an index of the relative balance between cartilage-associated and fibrotic collagen transcription.

The high-dose group provided an informative internal comparison. Although 100 J/cm^2^ did not cause extensive cytotoxicity, it failed to maintain the proliferative and chondrogenic advantages observed at 50 J/cm^2^ and was associated with increased expression of several undesirable markers. The reduced *Col2a1/Col1a1* ratio further indicates that the high-dose response involved a shift toward a less cartilage-specific and more fibrotic transcriptional profile rather than simply a reduction in cell number or bulk matrix production. Thus, increasing the irradiation condition beyond the best-performing tested exposure did not further enhance chondrogenesis but instead weakened phenotypic specificity.

Taken together with Fig. 3, these findings indicate that 50 J/cm^2^ irradiation improved the phenotypic specificity of ADSC chondrogenesis. Here, phenotypic specificity refers to the simultaneous enhancement of cartilage-associated matrix production and suppression of fibrotic, hypertrophic, and catabolic markers under the tested culture conditions; it does not imply permanent epigenetic reprogramming after irradiation withdrawal. Within this boundary, the data support the conclusion that intermediate-dose PBM improves both the magnitude and specificity of ADSC chondrogenesis.

### Repeated 940-nm PBM conditioning improves cell performance and cartilage formation within 3D porous scaffolds

Having established the effects of 940-nm PBM in pellet cultures, we next examined whether this response could be reproduced in a 3D tissue-engineering setting [25]. Egg white was selected as the scaffold precursor because it is inexpensive, widely available, and can be processed into a porous construct through a simple sequence of shaping, freeze drying, and thermal treatment, without the need for additional synthetic polymers or chemical cross-linking reagents [26]. ADSCs were seeded into the resulting 3D porous scaffolds and subjected to the indicated irradiation conditions (Fig. 5A). Gross examination showed that the scaffolds maintained a stable and well-defined shape, whereas scanning electron microscopy imaging confirmed a highly porous and interconnected architecture capable of supporting cell attachment and distribution throughout the construct (Fig. 5B).

**Fig. 5.**
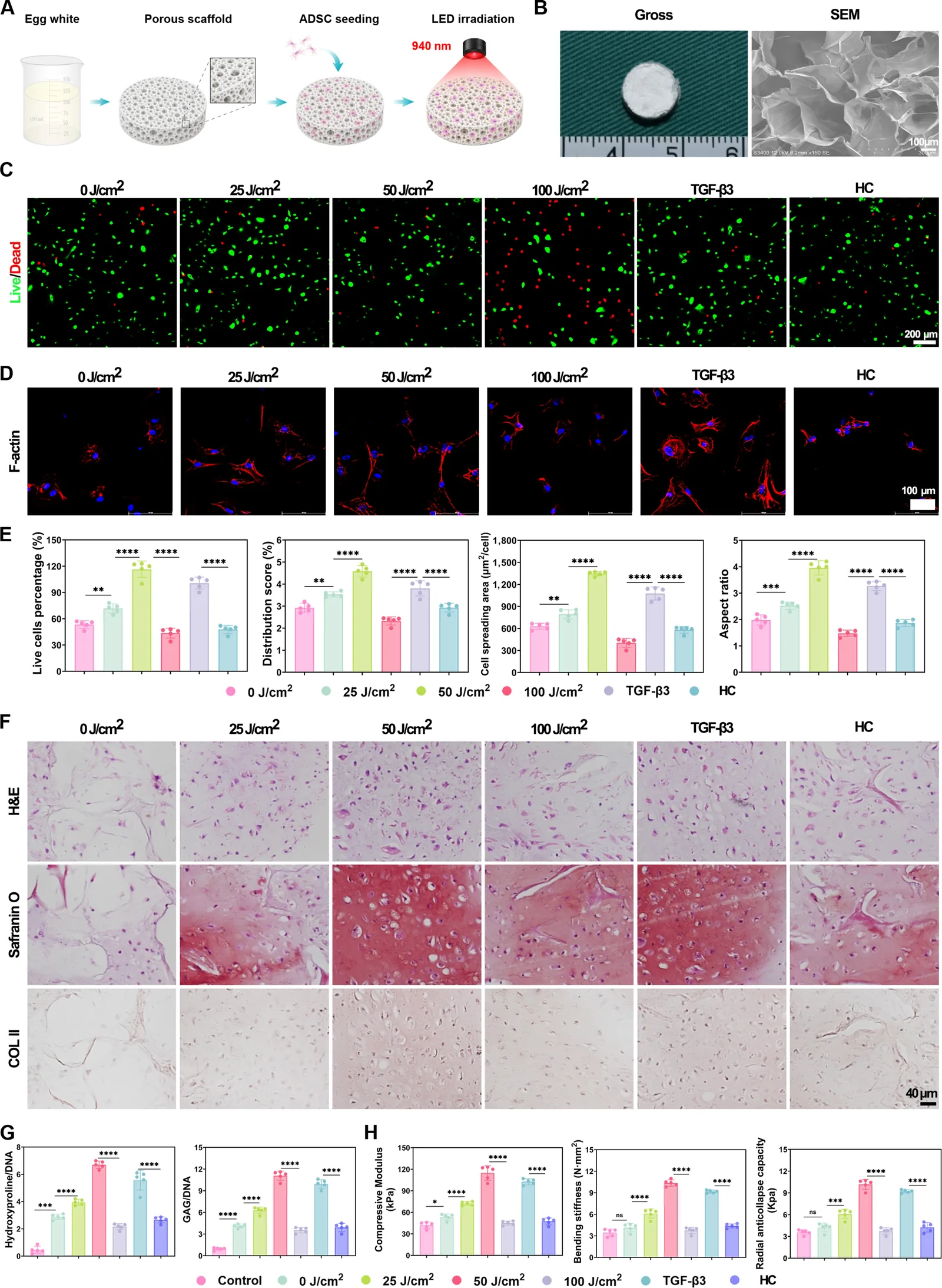
Photobiomodulation (PBM) at 940 nm improves adipose-derived stem cell (ADSC) performance and cartilage formation within porous scaffolds. (A) Schematic of egg white-derived porous scaffold fabrication, ADSC seeding, and 940-nm PBM. (B) Gross image and scanning electron microscopy (SEM) micrograph of the porous scaffold. (C) Representative Live/Dead staining images of ADSCs within the scaffolds after 5 d of the indicated treatments. Live cells, green; dead cells, red. (D) Representative F-actin staining showing ADSC attachment and spreading after 5 d of the indicated treatments. F-actin, red; nuclei, blue. (E) Quantification of live-cell percentage, cell-distribution score, spreading area, and aspect ratio. (F) Representative hematoxylin and eosin (H&E), Safranin O, and type II collagen (COL II) immunohistochemical staining of engineered constructs in the 0, 25, 50, and 100 J/cm^2^, transforming growth factor-β3 (TGF-β3), and heat control (HC) groups after 21 d of treatment. (G) Hydroxyproline/DNA and glycosaminoglycan (GAG)/DNA ratios. (H) Compressive modulus, bending stiffness, and radial anticollapse capacity of the constructs. The scaffold-only group without ADSCs is denoted Control. Data are presented as mean ± SD; *n* = 5 independent constructs derived from the same ADSC preparation. Statistical comparisons were performed by 1-way analysis of variance (ANOVA) followed by Tukey’s multiple-comparisons test. Horizontal brackets indicate the comparisons tested. ns, not significant; *P < 0.05; **P < 0.01; ***P < 0.001; ****P < 0.0001.

At day 5, Live/Dead staining revealed a high overall level of cell survival within the scaffolds, with the 50 J/cm^2^ group showing the greatest live-cell percentage and a more uniform cellular distribution (Fig. 5C and E). F-actin staining further showed improved spreading and cytoskeletal extension after 50 J/cm^2^ irradiation (Fig. 5D). Quantitative measurements of cell-distribution score, spreading area, and aspect ratio supported the imaging results (Fig. 5E).

These early changes are relevant because cellular behavior within a porous scaffold differs substantially from that on a 2D culture surface. Cells must attach to curved pore walls, migrate through interconnected spaces, and maintain sufficient metabolic activity despite spatial gradients in nutrients and oxygen [27]. The improved distribution and spreading observed after 50 J/cm^2^ preconditioning suggest that irradiation increased the capacity of ADSCs to adapt to this 3D microenvironment.

After 21 d, engineered constructs exposed to 50 J/cm^2^ showed more abundant tissue formation, stronger Safranin O staining, and increased COL II immunoreactivity (Fig. 5F). The increase in hydroxyproline/DNA and GAG/DNA ratios further indicated enhanced matrix deposition relative to cell content (Fig. 5G). These findings reproduced, within a scaffold environment, the matrix-producing phenotype observed in pellets.

The biological improvement was accompanied by improved mechanical properties of the engineered constructs resulting from combined scaffold support and tissue remodeling. Compressive modulus, bending stiffness, and radial anticollapse capacity were increased in the 50 J/cm^2^ group compared with untreated ADSC-loaded constructs and the heat-control group (Fig. 5H). This relationship between matrix formation and mechanical performance is central to the intended application. Tracheal cartilage must resist radial collapse while retaining sufficient flexibility to accommodate respiratory motion [28]. The combined improvement in matrix content, stiffness, and anticollapse behavior therefore represents a more meaningful endpoint than histological staining alone.

The mechanical performance of the engineered constructs reflects contributions from both the scaffold and the developing tissue. The acellular scaffold control demonstrated that the egg white-derived porous matrix provided intrinsic structural support, whereas ADSC incorporation enabled progressive ECM deposition and tissue remodeling. PBM conditioning further enhanced cell distribution, matrix accumulation, and construct-level mechanical performance. Thus, the scaffold should not be considered a passive cell carrier; rather, it provides a 3D structural and remodeling platform within which scaffold architecture, cell-derived matrix, and PBM-modulated ADSC activity jointly determine construct function.

### 940-nm PBM coordinates mitochondrial architecture, bioenergetics, and redox balance

We next investigated the intracellular processes associated with the best-performing chondrogenic response. Because PBM is frequently linked to mitochondrial regulation [29], we examined mitochondrial morphology, membrane potential, energy production, redox status, and respiratory function.

MitoTracker imaging showed marked differences in mitochondrial organization among the irradiation groups (Fig. 6A). Mitochondria in the 50 J/cm^2^ group formed longer and more highly branched networks, whereas networks in the untreated and high-dose groups were comparatively fragmented or less interconnected. Quantification of network length, branch points, and network score confirmed that mitochondrial architecture was most extensively reorganized under the intermediate-dose condition (Fig. 6B).

**Fig. 6.**
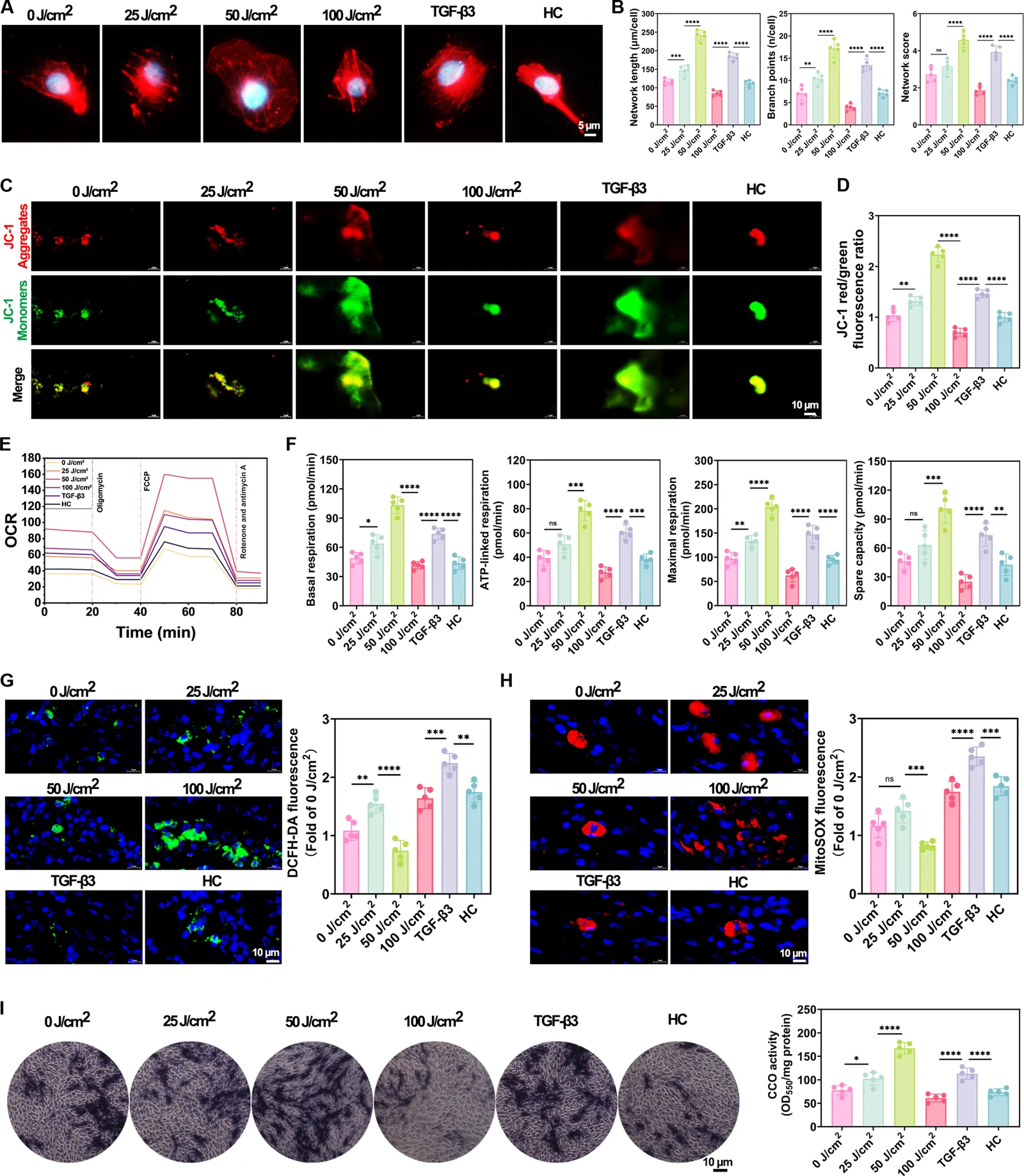
Photobiomodulation (PBM) at 940 nm remodels mitochondrial architecture, bioenergetics, and redox status in adipose-derived stem cells (ADSCs). (A) Representative MitoTracker-labeled mitochondrial networks in the 0, 25, 50, and 100 J/cm^2^, transforming growth factor-β3 (TGF-β3), and heat control (HC) groups after 21 d of the indicated treatments. Mitochondria, red; nuclei, blue. (B) Quantification of mitochondrial network length, branch points, and network score. (C) Representative JC-1 aggregate, monomer, and merged images used to assess mitochondrial membrane potential. JC-1 aggregates, red; JC-1 monomers, green. (D) Quantification of the JC-1 red-to-green fluorescence ratio. (E) Oxygen-consumption-rate (OCR) profiles during the sequential addition of oligomycin, carbonyl cyanide-p-trifluoromethoxyphenylhydrazone (FCCP), and rotenone plus antimycin A. (F) Basal respiration, adenosine triphosphate (ATP)-linked respiration, maximal respiration, and spare respiratory capacity derived from the OCR profiles. (G) Representative 2′,7′-dichlorodihydrofluorescein diacetate (DCFH-DA) fluorescence images and quantification of intracellular reactive oxygen species (ROS). DCFH-DA, green; nuclei, blue. (H) Representative MitoSOX fluorescence images and quantification of mitochondrial superoxide. MitoSOX, red; nuclei, blue. (I) Representative cytochrome-c oxidase (CCO) activity staining and quantitative analysis of CCO activity. Data are presented as mean ± SD; *n* = 5 independent culture-level experimental units derived from the same ADSC preparation. Statistical comparisons were performed by 1-way analysis of variance (ANOVA) followed by Tukey’s multiple-comparisons test; OCR time courses were analyzed by 2-way ANOVA. Horizontal brackets indicate the comparisons tested. ns, not significant; *P < 0.05; **P < 0.01; ***P < 0.001; ****P < 0.0001.

Mitochondrial morphology is dynamically linked to metabolic capacity and stress adaptation [30]. Increased network connectivity can facilitate distribution of metabolites and membrane potential, although morphology alone cannot establish improved mitochondrial function [31]. We therefore evaluated complementary bioenergetic endpoints.

JC-1 fluorescence was used to assess mitochondrial membrane potential [32]. In polarized mitochondria with a relatively high membrane potential, JC-1 accumulates within the mitochondrial matrix and forms red-fluorescent aggregates. By contrast, when the mitochondrial membrane potential is reduced, JC-1 remains predominantly in its green-fluorescent monomeric form [33]. Accordingly, the relative abundance of red aggregates versus green monomers provides a semiquantitative indicator of mitochondrial polarization. Representative images showed a more pronounced aggregate-dominant fluorescence pattern in the 50 J/cm^2^ group than in the untreated, 100 J/cm^2^, and HC groups (Fig. 6C). Quantitative analysis confirmed that the red-to-green fluorescence ratio was highest after 50 J/cm^2^ irradiation (Fig. 6D), indicating an increase in mitochondrial membrane potential. Cellular ATP content was also elevated under this condition (Fig. S6A and B). Seahorse analysis provided functional confirmation (Fig. 6E), showing that 50 J/cm^2^ irradiation increased basal respiration, ATP-linked respiration, maximal respiration, and spare respiratory capacity (Fig. 6F). Thus, the irradiation-induced remodeling of mitochondrial morphology was accompanied by enhanced mitochondrial polarization, oxidative phosphorylation, and bioenergetic reserve.

The redox measurements revealed a more nuanced response. The best-performing group did not exhibit the greatest accumulation of either intracellular or mitochondrial ROS. Instead, 2′,7′-dichlorodihydrofluorescein diacetate (DCFH-DA) and MitoSOX measurements indicated that excessive ROS accumulation was more evident under non-best-performing conditions, particularly after high-dose irradiation (Fig. 6G and H). This pattern suggests that the beneficial response cannot be explained by a simple linear increase in ROS. Rather, 50 J/cm^2^ irradiation combined enhanced respiration and cellular ATP content with restrained oxidative burden.

This distinction is important because ROS can function both as second messengers and as mediators of oxidative damage. A transient or spatially restricted redox signal may participate in pathway activation without producing a sustained increase in bulk ROS fluorescence [34]. Conversely, excessive mitochondrial ROS can damage proteins, lipids, and mitochondrial components, thereby limiting differentiation. The current data therefore support the concept of redox remodeling rather than indiscriminate ROS generation.

CCO activity also increased after 50 J/cm^2^ irradiation (Fig. 6I). This finding is compatible with enhanced respiratory-chain activity and may contribute to the bioenergetic response. However, increased CCO activity after irradiation does not by itself establish CCO as the primary photoreceptor for 940-nm light. CCO activation may represent a downstream consequence of altered calcium or mitochondrial state, or it may operate as a parallel component of the response [35]. Accordingly, we interpret the CCO measurements as evidence of respiratory-chain engagement rather than definitive proof of the initiating photoreceptive event.

Together, these results indicate that the best-performing radiant exposure establishes a mitochondrial condition characterized by network remodeling, increased membrane potential, enhanced respiratory reserve and ATP availability, and controlled rather than excessive oxidative stress. This bioenergetic–redox configuration provides a plausible intracellular context for the sustained biosynthetic demands of cartilage-matrix production.

### TRPV1-associated Ca^2+^ signaling and the Akt/GSK3β–SOX9 axis contribute to the chondrogenic response

We next examined how the extracellular optical input might be coupled to the intracellular chondrogenic program. Based on the capacity of TRPV1 to conduct Ca^2+^ and translate physical stimuli into intracellular signaling [36], we evaluated the effects of TRPV1 inhibition, calcium chelation, ROS scavenging, and Akt inhibition under the 50 J/cm^2^ condition (Fig. 7A).

**Fig. 7.**
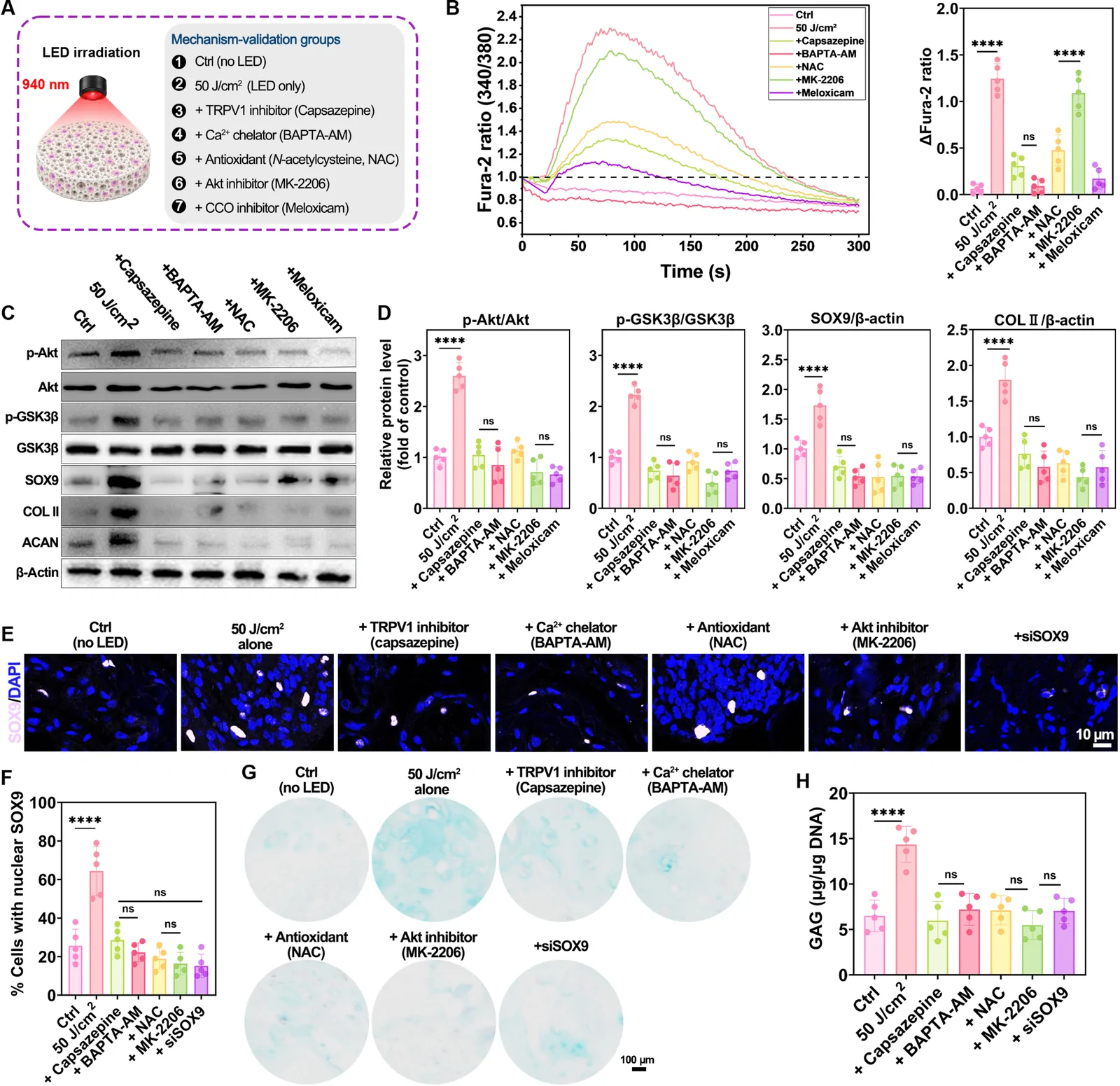
Pathway perturbation implicates transient receptor potential vanilloid 1 (TRPV1)-associated Ca^2+^ signaling and Akt/glycogen synthase kinase 3β (GSK3β)–SRY-box transcription factor 9 (SOX9) regulation in photobiomodulation (PBM)-enhanced adipose-derived stem cell (ADSC) chondrogenesis. (A) Experimental design for pathway interrogation at 50 J/cm^2^ using the TRPV1 inhibitor capsazepine, the intracellular Ca^2+^ chelator BAPTA acetoxymethyl ester (BAPTA-AM), the antioxidant N-acetylcysteine (NAC), the Akt inhibitor MK-2206, and a meloxicam comparison arm. A no-light-emitting diode (LED) group served as the control (Ctrl). (B) Fura-2 340/380 fluorescence-ratio traces and peak-minus-baseline Ca^2+^ responses after LED exposure. (C) Immunoblot analysis of phosphorylated and total Akt, phosphorylated and total GSK3β, SOX9, type II collagen (COL II), and ACAN, with β-actin as the loading control. (D) Densitometric quantification of p-Akt/Akt, p-GSK3β/GSK3β, SOX9/β-actin, and COL II/β-actin. (E) Representative SOX9 immunofluorescence images after pharmacological inhibition or SOX9 knockdown. The groups were Ctrl, 50 J/cm^2^, TRPV1 inhibitor (capsazepine), Ca^2+^ chelator (BAPTA-AM), antioxidant (NAC), Akt inhibitor (MK-2206), and siSOX9. All intervention groups received 50 J/cm^2^ irradiation unless otherwise indicated. SOX9, magenta; 4′,6-diamidino-2-phenylindole (DAPI), blue. (F) Percentage of cells exhibiting nuclear SOX9 localization. (G) Representative Alcian blue staining after the indicated interventions. (H) Glycosaminoglycan (GAG) content normalized to DNA. Data are presented as mean ± SD; *n* = 5 independent culture-level experimental units derived from the same ADSC preparation. Ca^2+^ time courses were analyzed by 2-way analysis of variance (ANOVA), whereas endpoint data were analyzed by 1-way ANOVA followed by Tukey’s multiple-comparisons test. Horizontal brackets indicate the comparisons tested. ns, not significant; ****P < 0.0001.

Fura-2 imaging showed that 940-nm PBM induced a rapid increase in intracellular Ca^2+^ (Fig. 7B). This response was attenuated by capsazepine and was strongly reduced by BAPTA acetoxymethyl ester (BAPTA-AM), supporting the involvement of TRPV1-associated Ca^2+^ signaling. These findings indicate that TRPV1 activity contributes to the irradiation-associated calcium response. They do not demonstrate that TRPV1 directly absorbs 940-nm photons; instead, they establish that TRPV1-dependent Ca^2+^ entry is required for efficient propagation of the cellular response.

Immunoblot analysis showed that 50 J/cm^2^ irradiation increased Akt phosphorylation at Ser473 and GSK3β phosphorylation at Ser9 (Fig. 7C and D). Because Ser9 phosphorylation inhibits GSK3β activity [37], these changes are consistent with activation of an Akt-dependent signaling configuration favorable to SOX9 stability and function. SOX9, COL II, and ACAN abundance increased in parallel.

Interference at different levels of the proposed pathway weakened these responses. Capsazepine treatment reduced Akt and GSK3β phosphorylation and decreased SOX9 and cartilage-matrix proteins. BAPTA-AM produced a similar attenuation, consistent with Ca^2+^ acting downstream of TRPV1-associated signaling. N-acetylcysteine (NAC) also reduced the chondrogenic response, indicating that redox signaling contributed to signal transmission even though the best-performing tested condition did not produce the highest steady-state ROS accumulation. This apparent difference can be reconciled if a limited redox signal is necessary for pathway activation, whereas excessive ROS accumulation reflects loss of redox control.

MK-2206 reduced Akt pathway activation and downstream SOX9 and matrix-associated proteins, while SOX9 knockdown directly impaired the final transcriptional and functional outputs. SOX9 immunofluorescence showed pronounced nuclear localization after 50 J/cm^2^ irradiation, which was reduced following TRPV1 inhibition, calcium chelation, antioxidant treatment, Akt inhibition, or SOX9 silencing (Fig. 7E and F). Alcian blue staining and GAG/DNA measurements showed corresponding reductions in matrix synthesis (Fig. 7G and H). SOX9 mRNA expression also declined after interruption of the pathway at these levels (Fig. S7). Meloxicam was included as a cyclooxygenase-sensitive pharmacological comparator rather than a pathway-specific probe. It was used to assess whether the irradiation-associated chondrogenic response could be explained by a general cyclooxygenase-dependent mechanism, and its effects were therefore not used to infer the causal ordering of the proposed TRPV1-associated Ca^2+^, redox, Akt/GSK3β, and SOX9 signaling components.

The concordance between calcium imaging, pathway phosphorylation, SOX9 localization, and GAG production supports a pathway-consistent signaling model rather than a set of unrelated associations. The data support a model in which TRPV1-dependent Ca^2+^ influx promotes NAC-sensitive Akt activation, leading to inhibitory Ser9 phosphorylation of GSK3β, enhanced SOX9 nuclear activity, and subsequent cartilage-matrix synthesis. The proposed pathway is summarized in Fig. S8.

Several qualifications are important. First, the inhibitor experiments establish functional dependence but do not prove that TRPV1 is itself the direct molecular chromophore for 940-nm PBM. Second, the present data do not fully resolve whether mitochondrial remodeling lies obligatorily downstream of TRPV1, because the complete mitochondrial phenotype was not measured after every intervention. Third, CCO activity was responsive to irradiation, but the current experiments do not distinguish whether this response is upstream, downstream, or parallel to TRPV1-dependent calcium signaling. We therefore describe 940-nm irradiation as coordinating TRPV1-dependent Ca^2+^ signaling and mitochondrial redox remodeling, rather than assigning a single exclusive photoreceptor.

### PBM conditioning preserves chondrogenic identity under inflammatory stress

The implantation environment contains inflammatory mediators that can destabilize cartilage-like tissue and promote matrix degradation [38]. We therefore tested whether the irradiation-associated phenotype persisted under IL-1β challenge (Fig. 8A).

**Fig. 8.**
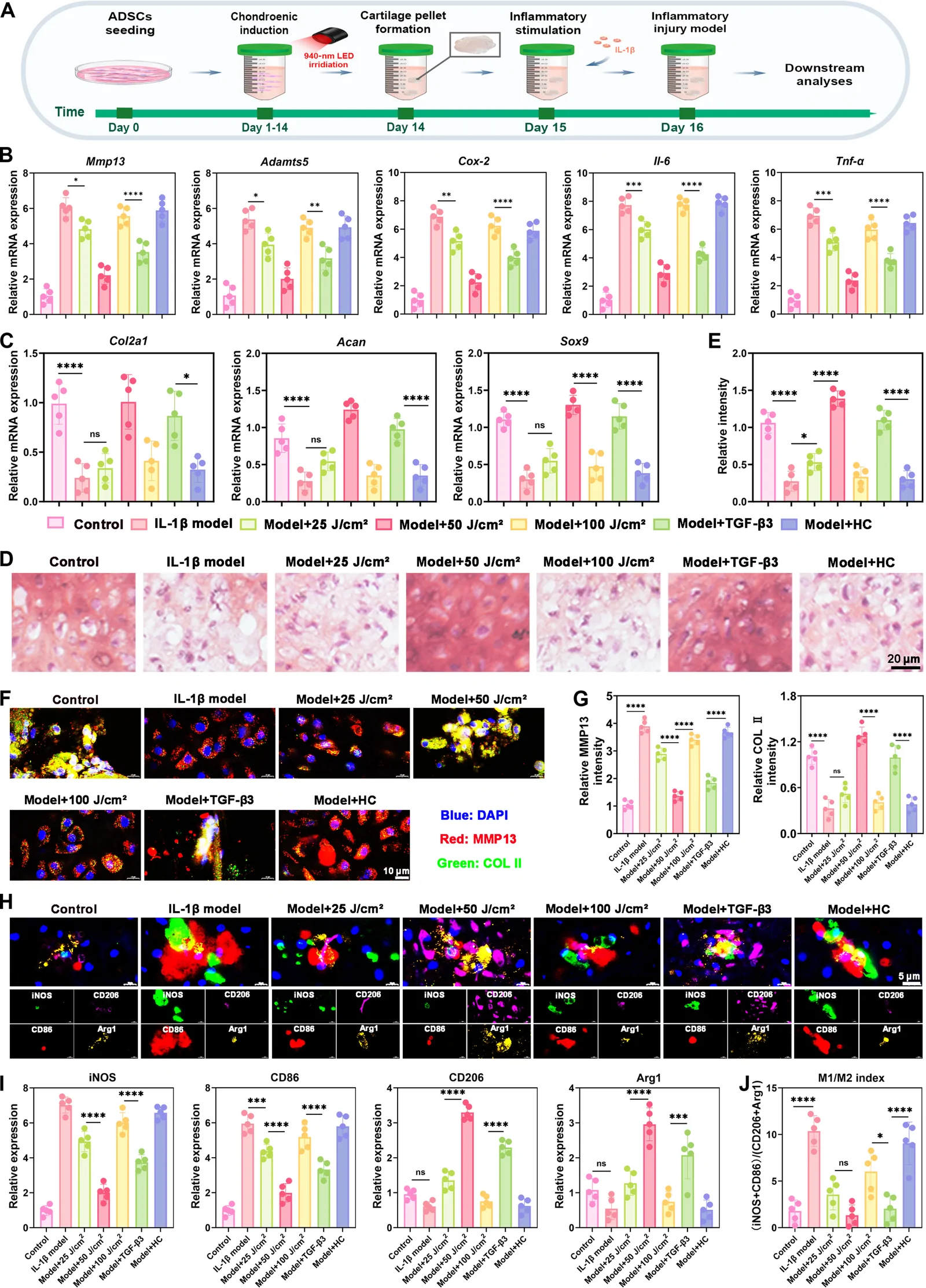
Photobiomodulation (PBM) at 940 nm preserves the chondrogenic phenotype under inflammatory challenge and alters macrophage-associated inflammatory marker profiles. (A) Timeline of adipose-derived stem cell (ADSC) pellet formation, chondrogenic induction with once-daily irradiation, interleukin-1 beta (IL-1β) challenge, and downstream analyses. (B) Reverse transcription quantitative polymerase chain reaction (RT-qPCR) analysis of the inflammatory and catabolic genes *Mmp13*, *Adamts5*, *Cox-2*, *Il-6*, and *Tnf-α* in the Control, IL-1β model, Model + 25 J/cm^2^, Model + 50 J/cm^2^, Model + 100 J/cm^2^, Model + transforming growth factor-β3 (TGF-β3), and Model + heat control (HC) groups. (C) RT-qPCR analysis of the chondrogenic genes *Col2a1*, *Acan*, and *Sox9* in the indicated groups. (D) Representative Safranin O staining of pellets after inflammatory challenge. (E) Quantification of Safranin O staining intensity. (F) Representative type II collagen (COL II) and matrix metallopeptidase 13 (MMP13) immunofluorescence images. COL II, green; MMP13, red; nuclei, blue. (G) Quantification of MMP13 and COL II fluorescence. (H) Representative immunofluorescence images of the M1-associated markers inducible nitric oxide synthase (iNOS) and CD86 and the M2-associated markers CD206 and Arg1. Nuclei were counterstained with 4′,6-diamidino-2-phenylindole (DAPI). (I) Quantification of iNOS, CD86, CD206, and Arg1 expression. (J) M1/M2 index calculated as (iNOS + CD86)/(CD206 + Arg1). Data are presented as means ± SD; *n* = 5 independent culture-level experimental units derived from the same ADSC preparation. Statistical comparisons were performed by 1-way analysis of variance (ANOVA) followed by Tukey’s multiple-comparisons test. Horizontal brackets indicate the comparisons tested. ns, not significant; *P < 0.05; **P < 0.01; ***P < 0.001; ****P < 0.0001.

IL-1β markedly increased the expression of *Mmp13*, *Adamts5*, *Cox-2*, *Il-6*, and *Tnf-α* genes, confirming establishment of an inflammatory and catabolic state (Fig. 8B). Preconditioning with 50 J/cm^2^ reduced these responses more effectively than the lower or higher irradiation conditions. At the same time, IL-1β suppressed the chondrogenic genes *Col2a1*, *Acan*, and *Sox9*, whereas 50 J/cm^2^ treatment partially or substantially restored their expression (Fig. 8C).

Histological analysis supported the transcriptional findings. IL-1β reduced Safranin O staining, consistent with loss of proteoglycan-rich matrix, while the 50 J/cm^2^ condition preserved substantially greater staining intensity (Fig. 8D and E). COL II/MMP13 immunofluorescence further showed that the 50 J/cm^2^ condition maintained an anabolic cartilage-associated marker while restricting a major catabolic enzyme (Fig. 8F and G).

These observations extend the significance of the earlier chondrogenesis experiments. Under standard induction conditions, 50 J/cm^2^ increased matrix formation and suppressed undesirable lineage programs. Under inflammatory challenge, the same dose helped preserve that phenotype. The response is therefore better described as increased phenotypic resilience than as an isolated increase in matrix synthesis.

We next assessed macrophage-associated inflammatory marker profiles using conditioned media from IL-1β-challenged pellets. Conditioned medium from pellets without light preconditioning increased the M1-associated markers inducible nitric oxide synthase (iNOS) and CD86 while reducing the M2-associated markers CD206 and Arg1 (Fig. 8H and I). In contrast, conditioned medium from pellets preconditioned at 50 J/cm^2^ decreased iNOS and CD86 and increased CD206 and Arg1, resulting in a lower M1/M2 marker index (Fig. 8J). These findings indicate that light preconditioning modified the paracrine inflammatory milieu generated by the ADSC pellets rather than directly inducing macrophage polarization. Thus, photonic preconditioning may influence not only the intrinsic chondrogenic state of ADSCs but also their capacity to shape the surrounding inflammatory environment [39]. However, the selected M1- and M2-associated markers provide only a simplified representation of macrophage heterogeneity [40]. Accordingly, these data should not be interpreted as evidence of complete polarization into discrete macrophage subtypes but rather as a shift in the relative balance between the measured inflammatory and reparative markers under the experimental conditions.

Overall, the inflammatory experiments identify a second functional consequence of the best-performing irradiation window. In addition to supporting matrix production, preconditioning limited inflammation-associated phenotypic erosion. This capacity is likely to be important after implantation, when regenerating cells are exposed simultaneously to cytokines, tissue injury, and mechanical stress.

### Preconditioned ADSC constructs improve early structural and functional tracheal repair in vivo

Finally, we evaluated whether the in vitro effects of 940-nm PBM translated into improved tracheal cartilage repair. ADSC-loaded circular scaffolds were exposed to the indicated irradiation conditions before implantation into rabbit tracheal cartilage defects, and repair was assessed 30 d after surgery (Fig. 9A).

**Fig. 9.**
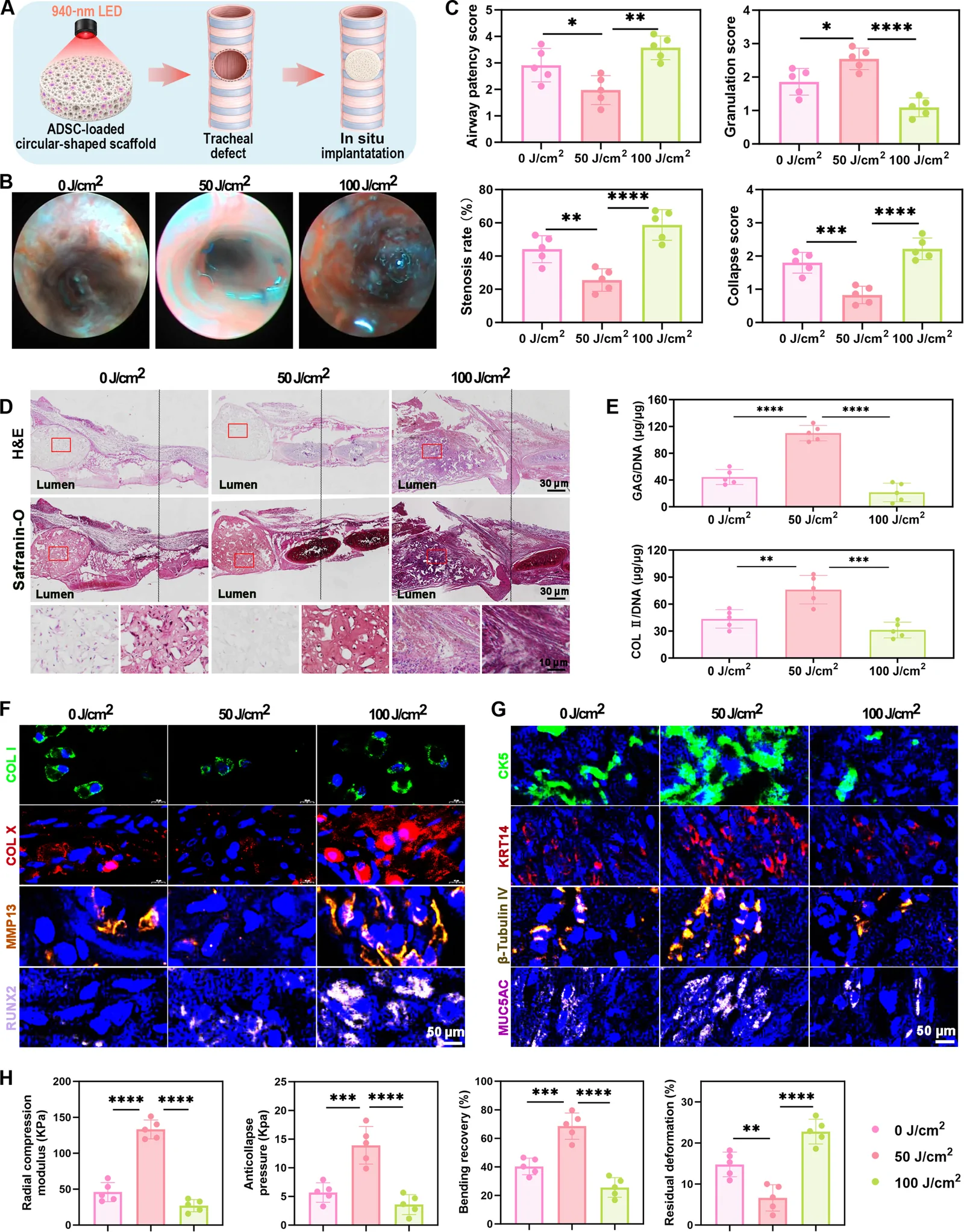
Photobiomodulation (PBM) at 940 nm adipose-derived stem cell (ADSC) constructs improve structural and functional repair of tracheal cartilage defects in vivo. (A) Schematic of 940-nm PBM of ADSC-loaded circular scaffolds and implantation into a tracheal cartilage defect in a rabbit model. Repair outcomes were evaluated 30 d after surgery. (B) Representative bronchoscopic images of repaired tracheae in the 0, 50, and 100 J/cm^2^ groups. (C) Airway patency, granulation, stenosis, and collapse scores. (D) Representative hematoxylin and eosin (H&E) and Safranin O staining of the repair site, with boxed regions shown at higher magnification. Black dashed lines separate the repaired segment from the adjacent native tracheal tissue. (E) Glycosaminoglycan (GAG)/DNA and type II collagen (COL II)/DNA ratios in repaired tissues. (F) Representative immunofluorescence images of type I collagen (COL I), type X collagen (COL X), matrix metallopeptidase 13 (MMP13), and runt-related transcription factor 2 (RUNX2) in the repair region. Nuclei were counterstained with 4′,6-diamidino-2-phenylindole (DAPI). (G) Representative immunofluorescence images of CK5, KRT14, β-tubulin IV, and MUC5AC used to evaluate epithelial repopulation and differentiation. Nuclei were counterstained with DAPI. (H) Radial compression modulus, anticollapse pressure, bending recovery, and residual deformation of the repaired tracheal segments. *n* = 5 independent animals per group. Bronchoscopic scores in (C) are presented as median with interquartile range and were analyzed using the Kruskal–Wallis test followed by Dunn’s multiple-comparisons test. Continuous quantitative outcomes in (E) and (H) are presented as means ± SD and were analyzed using 1-way analysis of variance (ANOVA) followed by Tukey’s multiple-comparisons test. Horizontal brackets indicate the comparisons tested. *P < 0.05; **P < 0.01; ***P < 0.001; ****P < 0.0001.

Bronchoscopic examination showed a more open and regular airway lumen in animals receiving constructs preconditioned at 50 J/cm^2^ than in those receiving constructs from the 0 or 100 J/cm^2^ groups (Fig. 9B). Correspondingly, the 50 J/cm^2^ group showed improved airway-patency scores and reduced granulation, stenosis, and collapse scores (Fig. 9C). These measurements indicate that the effect of preconditioning extended beyond tissue accumulation to the preservation of airway geometry.

Histological examination revealed more organized repair tissue and stronger Safranin O staining in the 50 J/cm^2^ group (Fig. 9D). Biochemical measurements further demonstrated increased GAG/DNA and COL II/DNA ratios (Fig. 9E). These findings are consistent with the formation of a cartilage-like ECM within the repaired segment.

The in vivo phenotype also recapitulated the suppression of maladaptive programs observed in vitro. COL I-, COL X-, MMP13-, and RUNX2-positive areas were reduced in the 50 J/cm^2^ group relative to the 0 and 100 J/cm^2^ groups (Fig. 9F and Fig. S9). Thus, the best-performing preconditioning condition not only increased cartilage-associated matrix but also limited fibrotic, hypertrophic, and catabolic features within the repair tissue.

Airway epithelial recovery was assessed using cytokeratin 5 (CK5), keratin 14 (KRT14), β-tubulin IV, and mucin 5AC (MUC5AC). The 50 J/cm^2^ group showed increased signals for these markers within the repaired region compared with the other irradiation conditions (Fig. 9G and Fig. S10). CK5 and KRT14 identify basal or activated epithelial populations, β-tubulin IV indicates ciliated differentiation, and MUC5AC marks mucus-producing cells [41]. Collectively, these findings indicate greater epithelial coverage and differentiation at the tissue level. However, because lineage tracing or donor-specific cell labeling was not performed, the cellular origin of these epithelial populations cannot be determined. The observed epithelial recovery may therefore reflect host epithelial migration, differentiation of implanted cells, or indirect effects of an improved structural and local regenerative environment and should not be interpreted as evidence of direct epithelial differentiation of the preconditioned ADSCs.

Mechanical assessment provided direct evidence that the histological changes were functionally relevant. Constructs preconditioned at 50 J/cm^2^ produced repaired tracheal segments with higher radial compression modulus, greater anticollapse pressure, and improved bending recovery, together with reduced residual deformation (Fig. 9H). These endpoints are particularly important because airway cartilage must resist collapse while retaining flexibility during respiratory movement.

The in vivo findings therefore link the molecular and cellular effects of 940-nm PBM to organ-level outcomes. The same intermediate dose that enhanced SOX9-dependent matrix production, suppressed fibrotic and hypertrophic drift, improved scaffold mechanics, and preserved the phenotype during inflammatory challenge also produced the most favorable airway structure and mechanical function after implantation. By contrast, the 100 J/cm^2^ group performed less well across these levels, reinforcing the biological significance of the dose window.

The 30-d observation period represents early repair rather than evidence of long-term regeneration. In addition, the in vivo comparison did not independently resolve the contributions of the scaffold, host cells, and implanted ADSCs. In particular, the epithelial-marker analysis was not accompanied by lineage tracing or donor-specific labeling; therefore, the relative contributions of host epithelial migration, implanted-cell differentiation, and indirect effects of improved airway stability remain unresolved. The results therefore support improved early structural and functional repair but not complete or permanent restoration of native tracheal cartilage.

### Integrated interpretation

This study provides a mechanistically informed translational proof of concept that repeated 940-nm PBM can enhance ADSC regenerative competence across multiple biological scales. Among the tested irradiation conditions, 50 J/cm^2^ produced the most favorable response, promoting proliferation, cartilage-associated matrix production, and SOX9 activity while limiting fibrotic, hypertrophic, and catabolic phenotypes. These effects were retained within porous scaffolds, where improved cell distribution and matrix deposition were accompanied by enhanced construct-level mechanical performance. PBM conditioning also increased resistance to inflammatory phenotype loss, and implantation of conditioned ADSC–scaffold constructs was associated with improved early cartilage-like matrix formation, airway patency, epithelial recovery, and mechanical function.

The principal advance is therefore not the identification of a single signaling pathway or the observation that PBM increases chondrogenesis. Rather, the findings show that a defined and externally controllable optical intervention can be used during ex vivo culture to enhance ADSC regenerative competence and translate this effect from cellular phenotype to engineered-construct performance and early airway-level repair. Mechanistically, the data implicate TRPV1-associated Ca^2+^ signaling, mitochondrial bioenergetic and redox remodeling, and Akt/GSK3β–SOX9 regulation in this response. However, TRPV1 has not been established as the direct 940-nm photoreceptor, increased CCO activity does not identify CCO as the initiating chromophore, and the causal hierarchy between membrane-associated calcium signaling and mitochondrial remodeling remains unresolved. Pharmacological perturbation should therefore be interpreted as evidence of pathway involvement rather than proof of an exclusive linear signaling sequence.

The treatment window should likewise be interpreted within the specific irradiation protocol used here. Because irradiance was held constant, radiant exposure and exposure duration were experimentally coupled, while repeated daily treatment introduced cumulative-dose effects and longer irradiation was accompanied by greater thermal exposure. The temperature-matched control argues against mild heating as the principal explanation for the favorable 50 J/cm^2^ response, but the contribution of the greater thermal burden at 100 J/cm^2^ cannot be excluded. Thus, 50 J/cm^2^ represents the best-performing condition among those tested rather than a universal optimum. Irradiation timing is also likely to be important: PBM was initiated at the beginning of pellet chondrogenesis or shortly after scaffold seeding, so the present study defines an early-conditioning strategy and does not establish whether comparable effects would occur if PBM were initiated in partially differentiated spheroids. In addition, the persistence of the induced state after PBM withdrawal remains unknown. The current data cannot distinguish durable cellular reprogramming from a transient metabolically permissive state, making dose, timing and postirradiation durability important parameters for future optimization.

The egg white-derived scaffold should be considered an active structural component of the engineered graft rather than a passive carrier. The porous architecture provides a 3D framework for cell retention, attachment, spatial distribution, matrix deposition, and baseline mechanical support, whereas PBM primarily modulates the functional state of the seeded ADSCs. Progressive matrix accumulation couples these cellular and material contributions at the construct level. After implantation, scaffold support, conditioned-cell activity, newly deposited matrix, and host-tissue remodeling are likely to contribute jointly to the observed repair. Because the in vivo study did not include acellular scaffold-only or scaffold-free controls, the independent effects of the scaffold on inflammation, epithelial recovery, and repaired-airway mechanics cannot be resolved. Similarly, the increased CK5, KRT14, β-tubulin IV, and MUC5AC signals should be interpreted as tissue-level epithelial recovery rather than evidence of direct epithelial differentiation of implanted ADSCs.

From a translational perspective, the approach investigated here represents an ex vivo manufacturing strategy rather than direct PBM of the recipient airway. The envisioned product is an autologous ADSC–scaffold graft conditioned with repeated 940-nm PBM before implantation. In the present study, the construct-conditioning phase lasted 21 d after cell seeding; a clinical workflow would additionally require adipose-tissue harvest, ADSC isolation, and expansion, construct preparation, and release testing. The optical conditioning step is externally controllable and does not require addition of a soluble agonist during irradiation, but the overall manufacturing process is not reagent-free, and the present experiments do not establish superiority over conventional biochemical induction. Future studies should determine whether the conditioning period can be shortened and directly compare manufacturing efficiency, phenotype durability, safety, reproducibility, cost, and scalability.

Finally, the generalizability of the findings remains to be established. The study used ADSCs derived from subcutaneous adipose tissue of healthy adult female rabbits, and donor-dependent variability was not systematically evaluated. Age, sex, adipose-tissue depot, disease-associated cell state, and interdonor variability may influence both baseline chondrogenic competence and responsiveness to PBM. Moreover, the 30-d in vivo endpoint represents early repair rather than durable regeneration and does not establish long-term matrix maturation, scaffold remodeling, epithelial homeostasis, mechanical stability, or the absence of late stenotic, fibrotic, hypertrophic, or degenerative changes. Within these boundaries, the present findings support repeated 940-nm PBM conditioning as a controllable strategy for enhancing ADSC regenerative competence and provide a framework for refining its mechanistic basis and translational application.

## Conclusion

This study provides a mechanistically informed translational proof of concept that repeated 940-nm PBM can be used as an externally controllable physical conditioning strategy to enhance the regenerative competence of ADSCs. Within the tested dose range, 50 J/cm^2^ produced the most favorable response, promoting cartilage-associated matrix formation while limiting fibrotic, hypertrophic, and catabolic phenotypes, with corresponding improvements in ADSC–scaffold construct performance and early structural and functional tracheal repair. Mechanistic analyses support the involvement of TRPV1-associated Ca^2+^ signaling, mitochondrial bioenergetic and redox remodeling, and Akt/GSK3β–SOX9 regulation, although their precise causal hierarchy and the initiating photoreceptive event remain unresolved. Collectively, these findings support ex vivo 940-nm PBM conditioning as a promising approach for regulating ADSC state before implantation, while longer-term validation and further optimization of the manufacturing workflow are required before clinical translation.

## Materials and Methods

### Study design

The study was designed to determine whether 940-nm PBM enhanced the chondrogenic and regenerative competence of ADSCs and to investigate the cellular pathways associated with this response. The experiments were performed in 4 sequential stages. First, the effects of different radiant exposures on ADSC proliferation, cell-cycle progression, and viability were evaluated. Second, ADSC chondrogenesis was assessed in pellet cultures and porous scaffold constructs. Third, mitochondrial function and the TRPV1–Ca^2+^–Akt/GSK3β–SOX9 signaling pathway were examined. Finally, the capacity of PBM-conditioned constructs to resist inflammatory challenge and repair tracheal cartilage defects was evaluated.

Unless otherwise stated, cells were assigned to the following groups: 0 J/cm^2^, 25 J/cm^2^, 50 J/cm^2^, 100 J/cm^2^, TGF-β3, and HC. The 0 J/cm^2^ group underwent the same handling procedure but received no irradiation. The TGF-β3 group received 10 ng/ml recombinant TGF-β3 without irradiation. The HC group received no light and was subjected to a temperature profile matched to that of the 50 J/cm^2^ group.

For in vitro experiments, n denotes independently handled culture-level experimental units, including wells, pellets or scaffold constructs derived from the same ADSC preparation. Technical measurements and multiple imaging fields within the same experimental unit were averaged before statistical analysis. For the in vivo study, individual animals were the biological experimental units. The experimental groups, treatment schedules, assessment time points, and principal outcomes for each experimental module are summarized in Table S1.

In this study, radiant exposure refers to the optical energy delivered during each individual irradiation session, whereas cumulative exposure represents the sum of all daily irradiation sessions during the corresponding treatment period. The duration of irradiation treatment and the number of irradiation sessions are specified for each experimental module. Unless otherwise indicated, irradiation was discontinued before implantation, and no additional optical stimulation was applied after implantation.

### Isolation and culture of ADSCs

Primary rabbit ADSCs used for the in vitro and mechanistic experiments were isolated from subcutaneous adipose tissue harvested from a single healthy 12-month-old female New Zealand White rabbit weighing approximately 3.0 to 3.5 kg (Shanghai Jiagan Biotechnology Co., Ltd., China). All procedures were approved by the Institutional Animal Care and Use Committee of Shanghai Pulmonary Hospital (approval no. K21-355Y). All in vitro experiments were performed using this single ADSC preparation; cells were not pooled from multiple donors.

Adipose tissue was collected under sterile conditions, minced into fragments smaller than 1 mm^3^, and washed 3 times with phosphate-buffered saline (PBS; Gibco, Thermo Fisher Scientific). The tissue was digested in 0.1% type I collagenase (Sigma-Aldrich) at 37 °C for 45 min with gentle agitation. Digestion was terminated by adding an equal volume of low-glucose Dulbecco’s modified Eagle medium (DMEM-LG; Gibco) containing 10% fetal bovine serum (FBS; Gibco). The suspension was filtered through a 70-μm cell strainer and centrifuged at 300 × g for 5 min. The stromal vascular fraction was resuspended in complete growth medium consisting of DMEM-LG, 10% FBS, and 1% penicillin–streptomycin and seeded in tissue-culture flasks.

Cells were maintained at 37 °C in a humidified atmosphere containing 5% CO_2_. Nonadherent cells were removed after 24 h, and the medium was changed every 2 to 3 d. ADSCs were passaged at approximately 80% to 90% confluence using 0.25% trypsin–EDTA. Passage-3 to passage-5 cells were used for all experiments.

### Phenotypic characterization of ADSCs

ADSC morphology was documented using an inverted phase-contrast microscope (IX73, Olympus). For crystal violet staining, passage-3 cells were fixed with 4% paraformaldehyde for 15 min, washed with PBS, and stained with 0.1% crystal violet solution for 15 min.

For flow-cytometric characterization, ADSCs were detached using trypsin–EDTA, washed with PBS containing 1% bovine serum albumin (BSA; Gibco), and incubated for 30 min at 4 °C in the dark with species-reactive fluorophore-conjugated antibodies against CD29, CD44, CD73, CD105, CD34, and human leukocyte antigen-DR/major histocompatibility complex class II. Corresponding isotype-matched antibodies were used as controls. Samples were acquired using a BD LSRFortessa flow cytometer and analyzed using FlowJo software v10.8. Details of all primary and secondary antibodies, including their suppliers, applications, and working dilutions, are provided in Table S2.

Multilineage differentiation was assessed using commercially available adipogenic, osteogenic, and chondrogenic differentiation media (Cyagen Biosciences). Adipogenic differentiation was evaluated by Oil Red O staining, osteogenic differentiation by Alizarin Red S staining, and chondrogenic differentiation by Alcian blue staining. Images were acquired under identical exposure settings, and staining intensity was quantified using ImageJ.

### 940-nm PBM system and optical dosimetry

A custom-built 940-nm PBM array comprising 24 high-power infrared emitters (SFH 4725AS A01, ams OSRAM, Germany) arranged in a 4 × 6 configuration and driven by a programmable constant-current controller was used. The irradiance at the sample plane was measured at multiple positions using an optical power meter, and the driving current was adjusted to achieve a mean irradiance of 100 mW/cm^2^. The LED panel was mounted 10 cm above the sample surface and connected to a programmable power controller. The center wavelength was 940 nm, and the spectral output was verified using a fiber-optic spectrometer (Ocean Optics). Irradiance at the sample plane was measured before each experiment using an optical power meter equipped with a near-infrared sensor (PM100D with S120VC sensor, Thorlabs).

The 940-nm LED source had a central wavelength of 940 nm with a spectral bandwidth of approximately 30 nm (FWHM). The LED generated a broad-area divergent beam with relatively uniform illumination across the target region. Irradiance was measured at the sample plane using a calibrated optical power meter under the experimental geometry, with a fixed source-to-sample distance of 10 cm. Spatial irradiance variation within the illuminated area was maintained within approximately ± 10%. The illuminated field covered approximately 60 cm^2^, encompassing the culture plate surface. During irradiation, cells were exposed through approximately 2 to 3 mm of culture medium in standard flat-bottom tissue culture plates. Radiant exposure was calculated according to:

$$\begin{array}{c} \begin{array}{c} \begin{array}{c} \text{Radiant exposure}\left(J/{\mathrm{cm}}^{2}\right)=\text{irradiance}\left(W/{\mathrm{cm}}^{2}\right)\times \text{exposure time}\;\left(s\right) \end{array} \end{array} \end{array}$$ (1)

Unless otherwise stated, the irradiance was maintained at 100 mW/cm^2^. Accordingly, exposure times were 250 s for 25 J/cm^2^, 500 s for 50 J/cm^2^ and 1,000 s for 100 J/cm^2^. Irradiation was applied once daily. The 0 J/cm^2^ and TGF-β3 groups were sham handled for 500 s, matching the principal 50 J/cm^2^ treatment condition. The HC groups remained beneath an identical inactive LED device for the corresponding handling period.

In this study, irradiation doses of 25, 50, and 100 J/cm^2^ refer to the radiant exposure delivered during a single irradiation session. The cumulative optical exposure was calculated as the sum of all irradiation sessions during the treatment period. For the 50 J/cm^2^ condition, cumulative exposures reached 350, 700, and 1,050 J/cm^2^ after 7, 14, and 21 daily irradiation sessions, respectively.

Culture-medium volume, plate type, and source-to-sample distance were kept constant within each experiment. Irradiation was performed on a thermostatically controlled platform. Samples were shielded from ambient light, and adjacent groups were irradiated separately to prevent scattered-light exposure.

### Temperature monitoring and HC treatment

Culture-medium temperature was monitored using a calibrated miniature thermocouple positioned in a cell-free reference well containing the same volume of medium as the experimental wells. Temperature was recorded at baseline and at 1-min intervals during irradiation. The maximum temperature increase was calculated as:

$$\Delta T_{\max}=T_{\max}-T_{0}$$ (2)

For the HC group, a programmable heating platform was used to reproduce the temperature–time profile measured during 50 J/cm^2^ irradiation without light exposure. The 0 J/cm^2^ control was maintained under identical handling conditions without either irradiation or programmed heating. The complete irradiation parameters and definitions of the principal experimental groups are summarized in Table S3.

### Cell proliferation assay

ADSCs were seeded in 96-well plates at 3 × 10^3^ cells per well in 100 μl of complete growth medium and allowed to attach overnight. Cells were subjected once daily to the indicated irradiation conditions. The TGF-β3 group received 10 ng/ml TGF-β3 in complete growth medium.

Cell proliferation was assessed on days 1, 3, 5, and 7 using a CCK-8 assay (Dojindo). Ten microliters of CCK-8 reagent was added to each well and incubated for 2 h at 37 °C. Absorbance at 450 nm was measured using a SpectraMax i3x microplate reader.

### Ki67 immunofluorescence

ADSCs were seeded on glass coverslips in 24-well plates at 2 × 10^4^ cells per well. After 3 daily treatments, cells were fixed with 4% paraformaldehyde for 15 min, permeabilized with 0.2% Triton X-100 for 10 min, and blocked with 5% BSA for 1 h.

Cells were incubated overnight at 4 °C with an anti-Ki67 antibody (Abcam; 1:300), followed by an Alexa Fluor 488-conjugated secondary antibody (Invitrogen; 1:500) for 1 h. Nuclei were stained with 4′,6-diamidino-2-phenylindole (DAPI; Sigma-Aldrich). Five randomly selected fields were imaged per sample using a confocal microscope (LSM 900, Zeiss). The percentage of Ki67-positive nuclei was quantified using ImageJ.

### EdU incorporation assay

DNA synthesis was evaluated on day 3 using an EdU cell proliferation kit (Beyotime). ADSCs were incubated with 10 μM EdU for 2 h at 37 °C, fixed with 4% paraformaldehyde, and permeabilized with 0.3% Triton X-100. EdU was detected using the manufacturer’s click-reaction solution, and nuclei were counterstained with DAPI. The proportion of EdU-positive cells was calculated from at least 5 randomly selected fields per sample.

### Cell-cycle analysis

ADSCs were collected 24 h after the third irradiation. Approximately 5 × 10^5^ cells per sample were washed with ice-cold PBS and fixed by slowly adding precooled 70% ethanol while vortexing. Cells were fixed overnight at 4 °C.

After washing, cells were incubated with 100 μg/ml ribonuclease A at 37 °C for 30 min and stained with 50 μg/ml propidium iodide for 30 min in the dark. At least 20,000 singlet events were acquired using a BD LSRFortessa flow cytometer. Doublets were excluded using propidium iodide-area versus propidium iodide-width gating. The proportions of cells in G_0_/G_1_, S, and G_2_/M phases were calculated using the cell-cycle platform in FlowJo.

### Live/Dead staining of ADSCs

Cell viability was evaluated on day 3 using a calcein-AM/propidium iodide Live/Dead assay kit (Beyotime). Cells were incubated with 2 μM calcein-AM and 4 μM propidium iodide for 20 min at 37 °C. Live cells were detected by green fluorescence and dead cells by red fluorescence. The dead-cell percentage was calculated from 5 randomly selected fields per sample.

### Chondrogenic pellet culture

ADSC pellets were generated by transferring 5 × 10^5^ cells into 15-ml conical tubes, followed by centrifugation at 500 × g for 5 min. Pellets were maintained in 0.5 ml of chondrogenic basal medium consisting of high-glucose DMEM, 1% insulin–transferrin–selenium, 100 nM dexamethasone, 50 μg/ml L-ascorbic acid-2-phosphate, 40 μg/ml L-proline, 1 mM sodium pyruvate, and 1% penicillin–streptomycin.

The 0, 25, 50, and 100 J/cm^2^ and HC groups were cultured in chondrogenic basal medium without exogenous TGF-β3. The TGF-β3 chondrogenic-reference group received 10 ng/ml recombinant TGF-β3 (R&D Systems) and no irradiation. LED irradiation was applied once daily from day 1 to day 14. Medium was replaced every 2 d.

Pellets were collected on day 14 for gross imaging, wet-weight determination, histology, immunofluorescence, biochemical analysis, RT-qPCR, and immunoblotting.

### Gross assessment of ADSC pellets

Pellets were photographed using a stereomicroscope immediately after removal from culture medium. Pellet cross-sectional area was calculated from calibrated images using ImageJ. Wet weight was measured after gently removing surface liquid with sterile filter paper.

Macroscopic compactness was evaluated independently by 2 blinded observers using a 5-point scale based on structural integrity, surface smoothness, and resistance to deformation: 1, fragmented or irregular; 2, poorly compacted; 3, moderately compact; 4, compact with a smooth surface; and 5, highly compact and spherical. The mean of the 2 observers’ scores was used.

### Histological staining of pellets and constructs

Samples were fixed in 4% paraformaldehyde for 24 h, dehydrated through graded ethanol, embedded in paraffin, and sectioned at 5 μm. Sections were stained with H&E and Safranin O using standard procedures.

Images were acquired with an Olympus BX53 microscope under identical settings. Safranin O-positive area and integrated staining intensity were quantified using ImageJ after color deconvolution. Threshold values were established using control sections and applied unchanged to all images within the same experiment.

### Immunofluorescence and immunohistochemistry

Paraffin sections were deparaffinized, rehydrated, and subjected to heat-mediated antigen retrieval in sodium citrate buffer (pH 6.0). Sections were blocked with 5% BSA for 1 h and incubated overnight at 4 °C with primary antibodies against aggrecan (Abcam; 1:200), COL II (Abcam; 1:200), COL1A1 (Abcam; 1:200), COL10A1 (Abcam; 1:200), RUNX2 (Cell Signaling Technology; 1:200), or MMP13 (Abcam; 1:200).

For immunofluorescence, sections were incubated with Alexa Fluor 488-, Alexa Fluor 555- or Alexa Fluor 647-conjugated secondary antibodies at 1:500 for 1 h, and nuclei were counterstained with DAPI. For COL II immunohistochemistry, endogenous peroxidase was blocked with 3% hydrogen peroxide, followed by incubation with an horseradish peroxidase-conjugated secondary antibody and development using a 3,3'-diaminobenzidine substrate kit (Servicebio).

Fluorescence intensity or positive area was quantified from at least 5 randomly selected fields per sample by an investigator blinded to group allocation.

### Matrix biochemical assays

Pellets, scaffold constructs or repaired tissues were weighed and digested overnight at 60 °C in papain digestion buffer containing 125 μg/ml papain, 5 mM cysteine hydrochloride, 5 mM EDTA, and 100 mM phosphate buffer (pH 6.5).

GAG content was measured using a dimethylmethylene blue assay. Absorbance was measured at 525 nm, and concentrations were calculated using a chondroitin sulfate standard curve. Total collagen was assessed using a hydroxyproline assay kit (Sigma-Aldrich). COL II content was measured using a species-compatible COL II enzyme-linked immunosorbent assay kit (Elabscience). DNA content was measured using a Quant-iT PicoGreen dsDNA assay kit (Invitrogen).

Total matrix content was expressed per pellet, construct, or repaired-tissue sample, as appropriate. Collagen/DNA, COL II/DNA, GAG/DNA, and hydroxyproline/DNA ratios were calculated to normalize matrix production to cell content.

### RNA extraction and RT-qPCR

Total RNA was extracted using TRIzol reagent (Invitrogen). RNA concentration and purity were measured using a NanoDrop One spectrophotometer. One microgram of total RNA was reverse transcribed using the PrimeScript RT reagent kit with genomic DNA removal (Takara).

Quantitative PCR was performed using TB Green Premix Ex Taq II (Takara) on a QuantStudio 5 Real-Time PCR System. The amplification program consisted of 95 °C for 30 s, followed by 40 cycles of 95 °C for 5 s and 60 °C for 30 s. Melt-curve analysis was performed to confirm amplification specificity.

Target genes included *Sox9*, *Col2a1*, *Acan*, *Col1a1*, *Col10a1*, *Runx2*, *Mmp13*, *Adamts5*, *Cox-2*, *Il-6*, *Tnf-α*, *Nos2*, *Cd86*, *Cd206*, and *Arg1*. *Gapdh* was used as the reference gene. Relative expression was calculated using the 2^−ΔΔCt^ method. The rabbit-specific primer sequences used for RT-qPCR are provided in Table S4.

To assess the relative transcriptional balance between cartilage-associated and fibrotic collagen expression, the *Col2a1/Col1a1* ratio was additionally calculated for each sample from the corresponding RT-qPCR data. Target-gene abundance relative to *Gapdh* was expressed as 2^−ΔCt^, where ΔCt = Ct_target_ − Ct_Gapdh_. The *Col2a1/Col1a1* ratio was calculated as (2^−ΔCt^ Col2a1)/(2^−ΔCt^ Col1a1), equivalent to 2^[−(Ct*Col2a1* − Ct*Col1a1*)]^. This ratio was interpreted as a relative transcriptional index and not as a direct biochemical measurement of type II-to-type I collagen content.

### Immunoblotting

Cells, pellets, or constructs were lysed in radioimmunoprecipitation assay buffer supplemented with protease and phosphatase inhibitor cocktails (Roche). Protein concentrations were measured using a bicinchoninic acid protein assay kit (Thermo Fisher Scientific).

Equal amounts of protein, typically 20 to 30 μg per lane, were separated by sodium dodecyl sulfate-polyacrylamide gel electrophoresis and transferred to polyvinylidene fluoride membranes. Membranes were incubated overnight at 4 °C with primary antibodies against SOX9, COL II, ACAN, COL1A1, COL10A1, RUNX2, MMP13, Akt, phospho-Akt (Ser473), GSK3β, phospho-GSK3β (Ser9), and β-actin. Antibody-specific suppliers and working dilutions are provided in Table S2.

After incubation with horseradish peroxidase-conjugated secondary antibodies at 1:5,000, proteins were detected using enhanced chemiluminescence reagent. Uncropped membranes were imaged using a ChemiDoc MP system (Bio-Rad). Band intensity was quantified using Image Lab software and normalized to β-actin.

### Fabrication of egg white-derived porous scaffolds

Fresh pathogen-free chicken egg white was collected under sterile conditions and gently homogenized with an equal volume of sterile PBS. The mixture was centrifuged at 1,000 × g for 10 min to remove bubbles and insoluble material.

The egg white solution was cast into polytetrafluoroethylene molds to produce circular scaffolds 5 mm in diameter and approximately 2 mm thick. Samples were frozen at −80 °C overnight and lyophilized for 48 h. The porous scaffolds were thermally stabilized by exposure to saturated steam for 5 min.

Scaffolds were washed extensively with sterile PBS, lyophilized again and sterilized with ethylene oxide. Before cell seeding, scaffolds were incubated overnight in complete growth medium.

For scanning electron microscopy analysis, dry scaffolds were sputter-coated with gold and imaged using a Hitachi SU8010 scanning electron microscope at an accelerating voltage of 5 kV.

### Seeding and culture of ADSC-loaded scaffolds

Each scaffold was placed in an ultralow-attachment 24-well plate and seeded with 1 × 10^6^ ADSCs suspended in 50 μl medium. The cell suspension was applied dropwise to both surfaces of the scaffold. Constructs were incubated for 2 h to permit cell attachment before 1 ml medium was added.

Acellular scaffolds were used as the Control group. ADSC-loaded constructs were assigned to the 0, 25, 50, or 100 J/cm^2^, TGF-β3, or HC groups. After an initial 24-h attachment period, constructs were cultured in chondrogenic basal medium and irradiated once daily. The TGF-β3 group received 10 ng/ml TGF-β3 without irradiation. Medium was changed every 2 d.

Early cell survival and spreading were evaluated on day 5. Histological, biochemical, and mechanical analyses were performed on day 21.

### Live/Dead and F-actin staining in scaffolds

On day 5, constructs were incubated with calcein-AM and propidium iodide for 30 min and imaged using confocal z-stacks. Live-cell percentage was calculated from 3 depths and at least 5 fields per construct.

For F-actin staining, constructs were fixed in 4% paraformaldehyde, permeabilized with 0.3% Triton X-100 and stained with tetramethylrhodamine isothiocyanate-phalloidin (Invitrogen; 1:200) for 1 h. Nuclei were stained with DAPI. Cell spreading area and aspect ratio were quantified using ImageJ.

Cell distribution was scored from 1 to 5 according to the proportion of scaffold area occupied by cells and the uniformity of cell distribution. Scoring was performed independently by 2 blinded investigators.

### Mechanical testing of engineered constructs

Mechanical testing was performed on day 21 using a universal testing machine equipped with a 10-N load cell (Instron 5943). Samples were maintained in PBS at room temperature throughout testing. The mechanical-testing configurations and calculation parameters for the engineered constructs are summarized in Table S5.

For compressive testing, constructs were compressed perpendicular to their flat surface at 0.5 mm/min to 30% strain. The compressive modulus was calculated from the linear region of the stress–strain curve between 5% and 15% strain.

Bending stiffness was determined using a 3-point bending fixture with a support span of 4 mm and a loading rate of 0.5 mm/min. Stiffness was calculated from the initial linear slope of the force–displacement curve.

Radial anticollapse capacity was determined by lateral compression of the construct at 0.5 mm/min until its diameter was reduced by 50%. The maximum stress recorded at 50% diameter reduction was used as the radial anticollapse value.

### MitoTracker analysis

ADSCs were cultured under chondrogenic conditions and exposed daily to the indicated treatments. On day 21, cells were incubated with 100 nM MitoTracker Deep Red FM (Invitrogen) for 30 min at 37 °C. Cells were washed, fixed with 4% paraformaldehyde, and counterstained with DAPI.

Images were acquired using a confocal microscope under identical laser power and detector settings. Mitochondrial network length, branch points, and network score were quantified using the MiNA plugin for ImageJ. At least 30 cells from 5 independent cultures were analyzed per group.

### Mitochondrial membrane-potential assay

On day 21, mitochondrial membrane potential was measured using a JC-1 assay kit (Beyotime). Cells were incubated with JC-1 working solution for 20 min at 37 °C and washed twice with JC-1 buffer. JC-1 aggregates were detected in the red channel, and monomers were detected in the green channel. The red-to-green fluorescence ratio was calculated for each field.

### ATP assay

Cellular ATP content was measured on day 14 using an ATP bioluminescence assay kit (Beyotime). Samples were lysed in ATP-releasing buffer and centrifuged at 12,000 × g for 5 min at 4 °C. Luminescence was measured using a microplate reader and quantified against an ATP standard curve. ATP content was normalized to total protein determined by the bicinchoninic acid method and was also expressed relative to the 0 J/cm^2^ group.

### Intracellular and mitochondrial ROS measurements

On day 21, intracellular ROS was assessed using DCFH-DA (Beyotime). Cells were incubated with 10 μM DCFH-DA for 30 min at 37 °C in serum-free medium.

Mitochondrial superoxide was assessed using 5 μM MitoSOX Red (Invitrogen) for 10 min at 37 °C. Cells were washed 3 times and imaged immediately using identical settings. Mean fluorescence intensity was normalized to that of the 0 J/cm^2^ group.

### Seahorse mitochondrial stress test

On day 21, mitochondrial respiration was assessed using a Seahorse XFe96 Extracellular Flux Analyzer and the Seahorse XF Cell Mito Stress Test Kit (Agilent). ADSCs were seeded at 2 × 10^4^ cells per well in Seahorse microplates and subjected to the indicated treatment schedule.

Before analysis, cells were incubated for 1 h in bicarbonate-free assay medium containing 10 mM glucose, 2 mM glutamine, and 1 mM pyruvate. Oxygen-consumption rate was recorded before and after sequential injection of 1.5 μM oligomycin, 1.0 μM carbonyl cyanide-p-trifluoromethoxyphenylhydrazone, and 0.5 μM rotenone plus 0.5 μM antimycin A.

Basal respiration, ATP-linked respiration, maximal respiration, and spare respiratory capacity were calculated using Wave software. Data were normalized to total protein content per well.

### CCO activity assay

On day 21, CCO activity was assessed using a mitochondrial complex IV activity assay kit (Sigma-Aldrich). Samples were lysed according to the manufacturer’s protocol. The oxidation of reduced cytochrome c was monitored at 550 nm using a microplate reader. CCO activity was normalized to total protein and expressed relative to the 0 J/cm^2^ group.

An increase in CCO activity was interpreted as evidence of respiratory complex IV engagement and not, by itself, as proof that CCO was the direct primary photoreceptor for 940-nm irradiation.

### Intracellular Ca^2+^ imaging

ADSCs were seeded in glass-bottom dishes and loaded with 5 μM Fura-2 AM and 0.02% Pluronic F-127 in Hanks’ balanced salt solution for 30 min at 37 °C. Cells were washed and allowed to de-esterify for 20 min.

Fura-2 fluorescence was alternately excited at 340 and 380 nm, and emission was collected at 510 nm using an inverted fluorescence microscope equipped with a rapid-wavelength switching system. Baseline fluorescence was recorded before irradiation, followed by continuous acquisition during the onset of 50 J/cm^2^ irradiation. Images were collected every 2 s for 300 s. The Ca^2+^ response was expressed as the Fura-2 340/380 ratio, and the peak-minus-baseline ratio was calculated for each cell.

### Pathway-inhibition and siSOX9 experiments

Mechanism-validation experiments were performed using the following groups: Ctrl, 50 J/cm^2^ alone, 50 J/cm^2^ + capsazepine, 50 J/cm^2^ + BAPTA-AM, 50 J/cm^2^ + NAC, 50 J/cm^2^ + MK-2206, and 50 J/cm^2^ + meloxicam. A separate siSOX9 group was included for SOX9-localization and matrix-production experiments.

Capsazepine (Sigma-Aldrich) was used at 10 μM, BAPTA-AM (MedChemExpress) at 10 μM, NAC (Sigma-Aldrich) at 5 mM, MK-2206 (Selleck Chemicals) at 1 μM, and meloxicam (Sigma-Aldrich) at 10 μM. Capsazepine and BAPTA-AM were added 30 min before irradiation; NAC, MK-2206, and meloxicam were added 1 h before irradiation. Inhibitors were retained during irradiation and for 6 h afterward, after which the medium was replaced. Treatment was repeated with each daily irradiation.

Meloxicam was included as a cyclooxygenase-sensitive pharmacological comparison to assess whether the irradiation-associated phenotype could be explained by a general COX-related inflammatory mechanism. It was not interpreted as a specific inhibitor of mitochondrial respiration or TRPV1-dependent signaling.

For SOX9 knockdown, ADSCs were transfected with 50 nM SOX9-targeting small interfering RNA or nontargeting control small interfering RNA (GenePharma, Shanghai, China) using Lipofectamine RNAiMAX (Invitrogen) 48 h before the first irradiation. Transfection was repeated on day 7 for 14-d experiments. Knockdown efficiency was verified by RT-qPCR and immunoblotting.

Samples were collected for Ca^2+^ imaging immediately after irradiation and for pathway phosphorylation, SOX9 localization, Alcian blue staining, and GAG/DNA analysis at the indicated endpoints.

### SOX9 nuclear-localization analysis

Cells were fixed and incubated with an anti-SOX9 antibody (Cell Signaling Technology; 1:200), followed by an Alexa Fluor 555-conjugated secondary antibody (Invitrogen; 1:500).

A cell was classified as showing nuclear SOX9 localization when the mean nuclear fluorescence intensity exceeded the cytoplasmic signal and the predefined threshold established from the Ctrl group. At least 100 cells from 5 randomly selected fields were analyzed per biological replicate.

### In vitro inflammatory-injury model

ADSC pellets were generated and subjected to daily irradiation during a 14-d chondrogenic induction period. On day 15, mature pellets were challenged with 10 ng/ml recombinant IL-1β (R&D Systems) for 24 h. No additional irradiation was administered during the IL-1β challenge.

Groups were Control, IL-1β model, Model + 25 J/cm^2^, Model + 50 J/cm^2^, Model + 100 J/cm^2^, Model + TGF-β3, and Model + HC. The LED, TGF-β3, and HC interventions were applied during the preceding 14-d pellet-conditioning period.

On day 16, pellets were collected for RT-qPCR analysis of *Mmp13*, *Adamts5*, *Cox-2*, *Il-6*, *Tnf-α*, *Col2a1*, *Acan*, and *Sox9*. Safranin O staining was used to assess matrix retention, and COL II/MMP13 immunofluorescence was used to evaluate anabolic and catabolic phenotypes. The concentrations, suppliers, treatment timing, and experimental roles of the pharmacological, cytokine, and gene-silencing interventions are summarized in Table S6.

### Macrophage-response assay

Primary rabbit bone marrow-derived macrophages were generated by culturing bone marrow mononuclear cells for 7 d in RPMI 1640 supplemented with 10% FBS, 1% penicillin–streptomycin, and 20 ng/ml recombinant macrophage colony-stimulating factor (PeproTech).

Conditioned media were collected from the inflammatory pellet groups after the 24-h IL-1β challenge, centrifuged at 1,000 × g for 10 min, and mixed 1:1 with fresh macrophage medium. Macrophages were incubated with the conditioned media for 24 h.

Cells were fixed and stained with antibodies against iNOS, CD86, CD206, and Arg1 (Proteintech; 1:200; Table S2). Marker abundance was quantified by immunofluorescence. The M1/M2 index was calculated as:

$$M1/M2\;\mathrm{index}\;=\;\left(\text{iNOS}+\mathrm{CD}86\right)/\left(\mathrm{CD}206+\text{Arg1}\right)$$ (3)

This index was used as a relative summary of the selected markers and was not interpreted as evidence that macrophages existed exclusively as discrete M1 or M2 populations.

### Animals and experimental groups

Healthy 12-month-old female New Zealand White rabbits weighing approximately 3.0 to 3.5 kg were obtained from Shanghai Jiagan Biotechnology Co., Ltd. (China). All procedures were approved by the Institutional Animal Care and Use Committee of Shanghai Pulmonary Hospital (K21-355Y) and complied with institutional guidelines. Animals were randomly assigned to 3 groups: 0, 50, and 100 J/cm^2^, with 5 animals per group. ADSC-loaded scaffolds were cultured under chondrogenic conditions and preconditioned once daily with the corresponding irradiation dose for 21 d before implantation. For the in vivo study, autologous ADSCs were isolated from each recipient animal using the same isolation and expansion procedure described above before scaffold seeding.

Animals, samples and data files were coded by an investigator not involved in surgery or outcome assessment. Histological scoring, fluorescence quantification, and mechanical testing were performed by investigators blinded to group assignment.

### Rabbit tracheal cartilage-defect model

Rabbits were anesthetized with intramuscular ketamine at 35 mg/kg and xylazine at 5 mg/kg, followed by maintenance with 1% to 2% inhaled isoflurane. After sterile preparation, a midline cervical incision was made, and the strap muscles were separated to expose the cervical trachea.

A 5-mm circular tracheal window was created in the anterior wall of the trachea using a biopsy punch. An ADSC-loaded circular scaffold of matching dimensions was implanted into the defect and secured to the adjacent cartilage using interrupted 6-0 absorbable sutures.

The muscular and skin layers were closed separately. Animals received postoperative analgesia with buprenorphine at 0.03 mg/kg every 12 h for 48 h and prophylactic penicillin for 3 d. Respiratory status, body weight, wound healing, and general condition were monitored daily during the first postoperative week and at least 3 times weekly thereafter.

### Bronchoscopy and airway scoring

Bronchoscopic examination was performed 30 d after implantation under sedation using a flexible veterinary bronchoscope. Airway patency, granulation, stenosis, and dynamic collapse were independently scored by 2 observers blinded to treatment allocation using predefined 0 to 4 ordinal scales (Table S7). Interobserver agreement was assessed using weighted Cohen’s κ. The mean of the 2 observer scores was used as the final score for each animal. Bronchoscopic scores were treated as ordinal outcomes, presented as median with interquartile range, and analyzed using the Kruskal–Wallis test followed by Dunn’s multiple-comparisons test. Representative images were selected only after completion of the quantitative analysis.

### Tissue collection and histological assessment

Animals were euthanized on day 30 using an overdose of pentobarbital sodium. The repaired tracheal segment, including at least 1 native cartilage ring proximal and distal to the defect, was harvested.

One portion was used for biochemical and mechanical testing, and the remainder was fixed in 4% paraformaldehyde for 48 h. Samples were embedded in paraffin and sectioned longitudinally at 5 μm. H&E and Safranin O staining were performed. The boundary between repaired and adjacent native tracheal tissue was identified during analysis.

For in vivo immunofluorescence, sections were stained for COL I, COL X, MMP13, and RUNX2 to assess fibrotic, hypertrophic, and catabolic features, and for CK5, KRT14, β-tubulin IV, and MUC5AC to evaluate tissue-level epithelial recovery and differentiation. Nuclei were counterstained with DAPI.

Positive areas were quantified using identical thresholds applied to all groups. At least 5 nonoverlapping fields covering the central and peripheral regions of the repair site were analyzed per animal.

### Mechanical testing of repaired tracheal segments

Mechanical tests were performed on freshly harvested tracheal segments maintained in PBS. The corresponding mechanical-testing conditions and outcome definitions for the repaired tracheal segments are also summarized in Table S5.

For radial compression, tracheal segments were compressed transversely using a universal testing machine at 1 mm/min. The radial compression modulus was calculated from the linear portion of the stress–strain curve.

Anticollapse pressure was determined by connecting each tracheal segment to a sealed negative-pressure system. Intraluminal pressure was progressively reduced, and the pressure at which the lumen cross-sectional area decreased by 50% was recorded.

For bending recovery, segments were bent to a predefined angle for 30 s and released. Recovery was calculated as the percentage of the initial angle restored after 60 s.

Residual deformation was determined after transverse compression and calculated as:

$$\text{Residual deformation}\left(\%\right)=\left(D_{0}-D_{\text{recovered}}\right)/D_{0}\times 100\%$$ (4)

where *D*_0_ is the initial external diameter and *D*_recovered_ is the diameter after unloading and recovery.

### Statistical analysis

Statistical analyses were performed using GraphPad Prism 10. For in vitro experiments, the experimental unit was an independently handled culture unit, such as a well, pellet, or scaffold construct, derived from the same ADSC preparation. Multiple imaging fields or technical measurements obtained from the same experimental unit were averaged before statistical analysis and were not treated as independent replicates. For in vivo experiments, each animal was considered an independent biological replicate.

Data distribution and homogeneity of variance were assessed using the Shapiro–Wilk and Brown–Forsythe tests, respectively. Unless otherwise stated, continuous data are presented as mean ± SD. Comparisons among more than 2 groups were performed using 1-way analysis of variance (ANOVA) followed by Tukey’s multiple-comparisons test. Time-course data were analyzed using 2-way ANOVA, with repeated-measures analysis applied when the same experimental unit was followed over time, followed by multiplicity-adjusted post hoc comparisons. Ordinal outcomes, including macroscopic compactness and bronchoscopic scores, are presented as median with interquartile range and were analyzed using the Kruskal–Wallis test followed by Dunn’s multiple-comparisons test. All tests were 2-sided. *P* < 0.05 was considered statistically significant. Significance symbols were defined as ns, not significant; *P < 0.05; **P < 0.01; ***P < 0.001; and ****P < 0.0001.

## Ethical Approval

All animal procedures were approved by the Institutional Animal Care and Use Committee of Shanghai Pulmonary Hospital (approval no. K21-355Y) and were conducted in accordance with institutional guidelines.

## Data Availability

The datasets generated and/or analyzed during the current study are available from the corresponding author upon reasonable request.
