# Supplementary material for: 940-nm Photobiomodulation Enhances ADSC-Mediated Tracheal Cartilage Repair through TRPV1-Associated Calcium Signaling and Mitochondrial Redox Remodeling
Source: Research (Wash D C). 2026 Oct 6;9:1440. doi: 10.34133/research.1440 (PMC13639145; doi:10.34133/research.1440)
Supplement: Supplementary file 1 [file research.1440.f1.zip › Supplemental Materials.docx]

**Supporting Information**

**940-nm photobiomodulation enhances ADSC-mediated tracheal cartilage repair through TRPV1-associated calcium signaling and mitochondrial redox remodeling**

*Yong Xu^1^, Erji Gao^1^, Zhe-Sheng Chen^2^, Bo Tao^1*^*

^1^ Department of Thoracic Surgery, Shanghai Pulmonary Hospital, Tongji University School of Medicine, Shanghai 200443, China;

^2^ Department of Pharmaceutical Science, College of Pharmacy and Health Science, St John’s University, Queens, New York 11439, United States of America.

*^*^* Corresponding author: Dr. Bo Tao, Email: tbo0820@163.com.

**Supplementary Fig. 1 | Phenotypic characterization and multilineage differentiation of ADSCs.**

**(A)** Representative phase-contrast morphology of passage-3 ADSCs. **(B)** Crystal violet staining of passage-3 ADSCs. **(C)** Flow-cytometric analysis of CD29, CD44, CD73, CD105, CD34 and HLA-DR; black traces indicate isotype controls and red traces indicate ADSCs. **(D)** Quantification of marker-positive cells. **(E)** Oil Red O, Alizarin Red and Alcian blue staining after adipogenic, osteogenic and chondrogenic induction, respectively. **(F)** Quantification of lineage-specific staining relative to the corresponding undifferentiated control. Data are presented as mean ± s.d.; n = 5 biologically independent samples. Statistical comparisons in F were performed by one-way ANOVA followed by Tukey’s multiple-comparisons test. *****P* < 0.0001.

**Supplementary Fig. 2 | Temperature profiles during 940-nm PBM.**

**(A)** Culture-medium temperature recorded during irradiation at 25, 50 or 100 J/cm^2^ or under the heat-control condition (HC). **(B)** Maximum temperature increase (ΔTmax) in each group. The HC condition was temperature-matched to the 50 J/cm^2^ group without light exposure. Data are presented as mean ± s.d.; n = 5 independent irradiation measurements. Statistical comparisons were performed by one-way ANOVA followed by Tukey’s multiple-comparisons test. Horizontal brackets indicate the comparisons tested. ns, not significant; *****P* < 0.0001.

**Supplementary Fig. 3 | Cell-cycle distribution of ADSCs after 940-nm PBM.**

**(A)** Quantification of the percentages of ADSCs in the G_0_/G_1_, S and G_2_/M phases after treatment with 0, 25, 50 or 100 J/cm^2^, TGF-β3 or HC. **(B)** Quantification of S-phase cells. Data are presented as mean ± s.d.; n = 5 biologically independent cultures. Statistical comparisons in B were performed by one-way ANOVA followed by Tukey’s multiple-comparisons test. Horizontal brackets indicate the comparisons tested. ****P* < 0.001; *****P* < 0.0001.

**Supplementary Fig. 4 | Magnified histological appearance of ADSC pellets after chondrogenic induction.**

Representative high-magnification H&E and Safranin O images of ADSC pellets cultured for 14 days after treatment with 0, 25, 50 or 100 J/cm^2^ 940-nm LED irradiation, TGF-β3 or HC.

**Supplementary Fig. 5 | Relative *Col2a1/Col1a1* expression ratio during ADSC chondrogenesis after 940-nm PBM.**

The *Col2a1/Col1a1* relative expression ratio was calculated from the corresponding RT–qPCR measurements in ADSC pellets treated with 0, 25, 50 or 100 J/cm^2^ 940-nm PBM, TGF-β3 or HC. The ratio was used as a relative index of the transcriptional balance between cartilage-associated type II collagen and fibrotic type I collagen. Data are presented as mean ± s.d.; n = 5 independent culture-level experimental units derived from the same ADSC preparation, with individual points representing the corresponding units. Statistical comparisons were performed by one-way ANOVA followed by Tukey’s multiple-comparisons test. Horizontal brackets indicate the comparisons tested. ***P* < 0.01; ****P* < 0.001; *****P* < 0.0001.

**Supplementary Fig. 6 | Cellular ATP content in ADSCs after 940-nm PBM.**

**(A)** Cellular ATP content normalized to total protein. **(B)** Relative cellular ATP content normalized to the 0 J/cm² group. ADSCs were analyzed at day 14 after treatment with 0, 25, 50 or 100 J/cm^2^, TGF-β3 or HC. Data are presented as mean ± s.d.; n = 5 biologically independent cultures. Statistical comparisons were performed by one-way ANOVA followed by Tukey’s multiple-comparisons test. Horizontal brackets indicate the comparisons tested. ns, not significant; ****P* < 0.001; *****P* < 0.0001.

**Supplementary Fig. 7 | Perturbation of TRPV1-associated Ca^2+^, redox, Akt and SOX9 signaling attenuates the 940-nm PBM-associated chondrogenic transcriptional response.**

Relative *SOX9* mRNA expression in ADSCs maintained without LED irradiation (Ctrl), exposed to 50 J/cm^2^ 940-nm PBM alone, or exposed to 50 J/cm^2^ irradiation in the presence of capsazepine, BAPTA-AM, NAC, MK-2206, meloxicam or SOX9-targeting siRNA (siSOX9). Bars represent the mean ± s.d.; individual dots represent biologically independent samples (n = 5). Statistical significance was assessed by one-way ANOVA followed by Tukey’s multiple-comparisons test. ns, not significant; ***P* < 0.01.

**Supplementary Fig. 8 | Working model illustrating the proposed involvement of TRPV1-associated Ca^2+^ signaling, mitochondrial bioenergetic/redox remodeling and Akt/GSK3β–SOX9 regulation in 940-nm PBM-enhanced chondrogenesis.**

Repeated 50 J/cm^2^ 940-nm PBM is associated with TRPV1-dependent Ca^2+^ entry and mitochondrial bioenergetic/redox remodeling, together with increased Akt phosphorylation, inhibitory Ser9 phosphorylation of GSK3β and enhanced SOX9 nuclear localization. Capsazepine, BAPTA-AM, N-acetylcysteine (NAC), MK-2206 and siSOX9 indicate the intervention points used to examine the functional involvement of TRPV1-associated Ca^2+^ signaling, redox regulation, Akt activity and SOX9. These perturbation experiments support pathway involvement but do not establish TRPV1 as the direct photoreceptor for 940-nm light or demonstrate an exclusive linear signaling sequence. Enhanced SOX9 activity is associated with increased COL2A1 and ACAN expression and cartilage-matrix synthesis. Dashed arrows indicate proposed or functionally supported relationships that have not been established as direct causal or sequential events.

**Supplementary Fig. 9 | Quantification of fibrotic, hypertrophic and catabolic markers in repaired tracheal tissue.**

COL I-, COL X-, MMP13- and RUNX2-positive areas in tracheal repair tissues from the 0, 50 and 100 J/cm^2^ groups. Data are presented as mean ± s.d.; n = 5 independent animals per group. Statistical comparisons were performed by one-way ANOVA followed by Tukey’s multiple-comparisons test. Horizontal brackets indicate the comparisons tested. ns, not significant; **P* < 0.05; ***P* < 0.01; *****P* < 0.0001.

**Supplementary Fig. 10 | Quantification of epithelial repopulation and differentiation markers in repaired tracheal tissue.**

CK5-, KRT14-, β-tubulin IV- and MUC5AC-positive areas in tracheal repair tissues from the 0, 50 and 100 J/cm^2^ groups. Data are presented as mean ± s.d.; n = 5 independent animals per group. Statistical comparisons were performed by one-way ANOVA followed by Tukey’s multiple-comparisons test. Horizontal brackets indicate the comparisons tested. ****P* < 0.001; *****P* < 0.0001.

**Table S1. Experimental groups, treatment schedules and outcome-assessment time points.**

| **Experimental module** | **Groups** | **Treatment schedule** | **Assessment time** | **Principal readouts** |
| --- | --- | --- | --- | --- |
| **ADSC proliferation and viability** | 0, 25, 50 and 100 J/cm^2^; TGF-β3; HC | Once-daily treatment after overnight attachment | CCK-8: days 1, 3, 5 and 7; Ki67, EdU and Live/Dead: day 3; cell cycle: 24 h after the third irradiation | Cell growth, Ki67 positivity, EdU incorporation, G_0_/G_1_-S-G_2_/M distribution and dead-cell percentage |
| **Pellet chondrogenesis** | 0, 25, 50 and 100 J/cm^2^; TGF-β3; HC | 5 × 10^5^ ADSCs per pellet; once-daily treatment on days 1-14; medium replacement every 2 days | Day 14 | Gross area, wet weight, compactness, histology, cartilage ECM, biochemical matrix content, RT-qPCR and immunoblotting |
| **ADSC-loaded porous scaffolds** | Acellular scaffold control; 0, 25, 50 and 100 J/cm^2^; TGF-β3; HC | 1 × 10^6^ ADSCs per scaffold; 24-h attachment followed by once-daily treatment; medium replacement every 2 days | Cell survival/spreading: day 5; histology, matrix biochemistry and mechanics: day 21 | Live/Dead, F-actin, cell distribution, COL II/GAG/hydroxyproline and construct mechanics |
| **Mitochondrial phenotype** | 0, 25, 50 and 100 J/cm^2^; TGF-β3; HC | Once-daily treatment under chondrogenic conditions | ATP: day 14; mitochondrial morphology, membrane potential, OCR, ROS and CCO activity: day 21 | MitoTracker network metrics, JC-1 ratio, ATP, respiration, spare respiratory capacity, DCFH-DA, MitoSOX and CCO activity |
| **Pathway interrogation** | Ctrl; 50 J/cm^2^; 50 J/cm^2^ + capsazepine; + BAPTA-AM; + NAC; + MK-2206; + meloxicam; siSOX9 subset | Interventions administered relative to each 50 J/cm^2^ session as detailed in Table S6 | Ca^2+^ imaging: immediately during irradiation; signaling, SOX9 localization and matrix endpoints: day 14 unless otherwise indicated | Fura-2 340/380 ratio, Akt/GSK3β phosphorylation, SOX9 localization, COL II/ACAN, Alcian blue and GAG/DNA |
| **Inflammatory pellet challenge** | Control; IL-1β model; Model + 25, 50 or 100 J/cm^2^; Model + TGF-β3; Model + HC | Pellets preconditioned for 14 days; 10 ng/mL IL-1β added on day 15 for 24 h; no additional irradiation during IL-1β exposure | Day 16 | Inflammatory/catabolic and chondrogenic transcripts, Safranin O, COL II and MMP13 |
| **Macrophage response** | Conditioned media derived from the inflammatory pellet groups | Macrophages differentiated for 7 days; conditioned medium mixed 1:1 with fresh macrophage medium and applied for 24 h | After 24-h conditioned-medium exposure | iNOS, CD86, CD206 and Arg1; M1/M2 index = (iNOS + CD86)/(CD206 + Arg1) |
| **Rabbit tracheal repair** | 0, 50 and 100 J/cm^2^; n = 5 rabbits per group | ADSC-loaded scaffolds preconditioned once daily for 21 days before implantation into 5-mm tracheal cartilage defects | Postoperative day 30 | Bronchoscopy, histology, matrix biochemistry, epithelial repopulation and differentiation, and mechanical function. |

Ctrl denotes the no-LED control used in mechanistic experiments. “Control” in the scaffold-mechanics panel denotes an acellular scaffold, whereas “Control” in the inflammatory experiment denotes pellets without IL-1β challenge.

**Table S2. Primary and secondary antibodies and working dilutions.**

| **Target/reagent** | **Application** | **Supplier** | **Working dilution** |
| --- | --- | --- | --- |
| **CD29, CD44, CD73 and CD105** | Flow cytometry; ADSC-positive markers | BD Biosciences | Directly conjugated antibody; manufacturer-recommended test volume |
| **CD34 and HLA-DR/MHC class II** | Flow cytometry; ADSC-negative markers | BD Biosciences | Directly conjugated antibody; manufacturer-recommended test volume |
| **Ki67** | Immunofluorescence | Abcam | 1:300 |
| **Aggrecan** | Pellet immunofluorescence | Abcam | 1:200 |
| **COL II** | IF/IHC; immunoblotting | Abcam | 1:200 (IF/IHC); 1:500-1:1,000 (WB) |
| **ACAN** | Immunoblotting | Abcam | 1:500-1:1,000 |
| **COL1A1/COL I** | IF; immunoblotting | Abcam | 1:200 (IF); 1:500-1:1,000 (WB) |
| **COL10A1/COL X** | IF; immunoblotting | Abcam | 1:200 (IF); 1:500-1:1,000 (WB) |
| **RUNX2** | IF; immunoblotting | Cell Signaling Technology | 1:200 (IF); 1:500-1:1,000 (WB) |
| **MMP13** | IF; immunoblotting | Abcam | 1:200 (IF); 1:500-1:1,000 (WB) |
| **SOX9** | IF; immunoblotting | Cell Signaling Technology | 1:200 (IF); 1:500-1:1,000 (WB) |
| **Akt** | Immunoblotting | Cell Signaling Technology | 1:1,000 |
| **Phospho-Akt (Ser473)** | Immunoblotting | Cell Signaling Technology | 1:1,000 |
| **GSK3β** | Immunoblotting | Cell Signaling Technology | 1:1,000 |
| **Phospho-GSK3β (Ser9)** | Immunoblotting | Cell Signaling Technology | 1:1,000 |
| **β-actin** | Immunoblotting loading control | Proteintech | 1:1,000 |
| **iNOS and CD86** | Macrophage IF; M1-associated markers | Proteintech | 1:200 |
| **CD206 and Arg1** | Macrophage IF; M2-associated markers | Proteintech | 1:200 |
| **CK5 and KRT14** | Airway basal/activated epithelial markers | Proteintech | 1:200 |
| **β-tubulin IV** | Ciliated epithelial marker | Abcam | 1:200 |
| **MUC5AC** | Mucus-producing epithelial marker | Abcam | 1:200 |
| **Alexa Fluor 488-, 555- or 647-conjugated secondary antibodies** | Immunofluorescence | Invitrogen | 1:500 |
| **HRP-conjugated secondary antibodies** | WB/IHC | Cell Signaling Technology | 1:5,000 (WB); according to kit instructions (IHC) |

IF, immunofluorescence; IHC, immunohistochemistry; WB, immunoblotting. Antibody information was compiled from the original experimental records. Only reagents and working conditions actually used in this study are listed.

**Table S3. Single-session and cumulative radiant exposures of 940-nm PBM treatment protocols.**

| **Parameter** | **0 J/cm^2^** | **25 J/cm^2^** | **50 J/cm^2^** | **100 J/cm^2^** | **TGF-β3** | **Heat control (HC)** |
| --- | --- | --- | --- | --- | --- | --- |
| **Light condition** | LED off; sham handled | 940-nm LED | 940-nm LED | 940-nm LED | LED off; biochemical positive control | LED off; temperature-matched control |
| **Nominal peak wavelength** | No light | 940 nm | 940 nm | 940 nm | No light | No light |
| **Irradiation mode** | No irradiation | Continuous wave | Continuous wave | Continuous wave | No irradiation | No irradiation |
| **Irradiance at sample plane** | 0 mW/cm^2^ | 100 mW/cm^2^ | 100 mW/cm^2^ | 100 mW/cm^2^ | 0 mW/cm^2^ | 0 mW/cm^2^ |
| **Single-session radiant exposure** | 0 J/cm^2^ | 25 J/cm^2^ | 50 J/cm^2^ | 100 J/cm^2^ | 0 J/cm^2^ | 0 J/cm^2^ |
| **Exposure/heating duration per session** | 500 s sham handling; LED off | 250 s | 500 s | 1,000 s | No irradiation; handled in parallel | 500 s; temperature-time profile matched to the 50 J/cm^2^ group |
| **Source-to-sample distance** | 10 cm; same positioning | 10 cm | 10 cm | 10 cm | 10 cm; LED off | 10 cm; LED off |
| **Treatment schedule/frequency** | Once daily | Once daily | Once daily | Once daily | No irradiation; 10 ng/mL TGF-β3 replenished with each medium change | Once daily |
| **Cumulative radiant exposure after 3 daily sessions** | 0 J/cm^2^ | 75 J/cm^2^ | 150 J/cm^2^ | 300 J/cm^2^ | 0 J/cm^2^ | 0 J/cm^2^ |
| **Cumulative radiant exposure after 7 daily sessions** | 0 J/cm^2^ | 175 J/cm^2^ | 350 J/cm^2^ | 700 J/cm^2^ | 0 J/cm^2^ | 0 J/cm^2^ |
| **Cumulative radiant exposure after 14 daily sessions** | 0 J/cm^2^ | 350 J/cm^2^ | 700 J/cm^2^ | 1,400 J/cm^2^ | 0 J/cm^2^ | 0 J/cm^2^ |
| **Cumulative radiant exposure after 21 daily sessions** | 0 J/cm^2^ | 525 J/cm^2^ | 1,050 J/cm^2^ | 2,100 J/cm^2^ | 0 J/cm^2^ | 0 J/cm^2^ |
| **Temperature monitoring** | Yes | Yes | Yes | Yes | Yes | Yes |
| **Primary purpose** | Baseline control | Low-dose PBM | Intermediate-dose PBM | High-dose PBM | Reference chondrogenic induction | Distinguish light-dependent effects from mild heating |

Radiant exposure was calculated as irradiance × exposure time. The HC group reproduced the temperature-time profile of the 50 J/cm^2^ condition without optical exposure. Cumulative radiant exposures clarify dose accumulation across the principal 3-, 7-, 14- and 21-day protocols; HC and TGF-β3 groups received no optical fluence.

**Table S4. RT-qPCR primer sequences for rabbit target genes.**

| **Gene symbol** | **Common target name** | **Forward primer (5′-3′)** | **Reverse primer (5′-3′)** |
| --- | --- | --- | --- |
| ***Sox9*** | SOX9 | CACACGTCAAGCGACCCATGAA | TCTTCTCGCTCTCGTTCAGCAG |
| ***Col2a1*** | Type II collagen | ACAGCAGGTTCACCTATACCG | CCCACTACCGGTGTGTTC |
| ***Acan*** | Aggrecan | GAGGATGGCTTCCACCAGT | TGGGGTACCTGACAGTCTGA |
| ***Col1a1*** | Type I collagen | AGGTGCTGATGGCTCTCCT | GGACCACTTTCACCCTTGT |
| ***Col10a1*** | Type X collagen | TCTGTGAGCTCCATGATTGC | GCAGCATTACGACCCAAGATC |
| ***Runx2*** | RUNX2 | CCGCACGACAACCGCACCAT | CGCTCCGGCCCACAAATCTC |
| ***Mmp13*** | Matrix metallopeptidase 13 | GATGACCTGTCTGAGGAAGACC | GCATTTCTCGGAGCCTGTCAAC |
| ***Adamts5*** | ADAMTS5 | CCTGCCCACGATGACTTCAT | GGTAGCCCACTTTGTCCTTG |
| ***Cox-2*** | Cyclooxygenase-2 (COX-2) | CAGGCTGAAATCTACCCTCC | GCTCGGCTTCCAGTATTGAG |
| ***Il-6*** | Interleukin-6 | TTGCCAAGCCTTGTCTGAGAT | TTCTCCCCCAGGGAGTTCAC |
| ***Tnf-α*** | Tumor necrosis factor-α | ATGAGCACTGAAAGCATGATCCGG | GCAATGATCCCAAAGTAGACCTGCCC |
| ***Nos2*** | Inducible nitric oxide synthase (iNOS) | CCAAGGCCAACCGCGAGAAGATGAC | AGGGTACATGGTGGTGCCGCCAGAC |
| ***CD86*** | CD86 | ACTGCTCATCTATACACGGTTACC | GGAAACGTCGTACAGTTCTGTG |
| ***CD206*** | CD206 | CCTGGTGCTGCTGTTCTCTA | GGCATAGGAGTCCTGGTTCA |
| ***Arg1*** | Arginase 1 | CTCCAAGCCAAAGTCCTTAGAG | AGGAGCTGTCATTAGGGACATC |
| ***Gapdh*** | GAPDH | CCGCCTGGAGAAAGCTGCTAA | ACGACCTGGTCCTCGGTGTA |

Gapdh was used as the reference gene.

**Table S5. Mechanical-testing conditions for engineered constructs and repaired tracheal segments.**

| **Specimen/endpoint** | **Test configuration** | **Loading or measurement conditions** | **Reported outcome** |
| --- | --- | --- | --- |
| **Engineered construct, day 21** | Unconfined compression perpendicular to flat surface | Instron 5943, 10-N load cell; 0.5 mm/min to 30% strain; samples maintained in PBS | Compressive modulus from the 5-15% linear strain region |
| **Engineered construct, day 21** | Three-point bending | Support span 4 mm; loading rate 0.5 mm/min | Bending stiffness from the initial linear slope of the force-displacement curve |
| **Engineered construct, day 21** | Lateral/radial compression | 0.5 mm/min until diameter was reduced by 50% | Maximum stress at 50% diameter reduction |
| **Repaired tracheal segment, day 30** | Transverse radial compression | Fresh segment maintained in PBS; loading rate 1 mm/min | Radial compression modulus from the linear portion of the stress-strain curve |
| **Repaired tracheal segment, day 30** | Negative-pressure collapse test | Sealed pressure system; intraluminal pressure progressively reduced | Pressure at which luminal cross-sectional area decreased by 50% |
| **Repaired tracheal segment, day 30** | Bending recovery | Held at a predefined angle for 30 s and released; assessed after 60 s | Percentage recovery toward the initial configuration |
| **Repaired tracheal segment, day 30** | Residual deformation after transverse compression | External diameter before compression and after unloading/recovery | Residual deformation (%) = (D_0_ - Drecovered)/D_0_ × 100 |

All mechanical tests were performed on hydrated samples. D_0_ denotes the initial external diameter and D_recovered_ denotes the external diameter after unloading and recovery.

**Table S6. Pharmacological, cytokine and gene-silencing interventions.**

| **Intervention** | **Working concentration** | **Supplier** | **Timing and duration** | **Purpose/interpretation** |
| --- | --- | --- | --- | --- |
| **Recombinant TGF-β3** | 10 ng/mL | R&D Systems | Added at 10 ng/mL to complete growth medium in proliferation assays and to chondrogenic medium in pellet and scaffold experiments; replenished with each medium change; no LED irradiation. | Positive reference for chondrogenic induction |
| **Recombinant IL-1β** | 10 ng/mL | R&D Systems | Added on day 15 after 14 days of pellet preconditioning; 24-h exposure without additional irradiation | Inflammatory and catabolic challenge |
| **Macrophage colony-stimulating factor** | 20 ng/mL | PeproTech | Present during 7-day differentiation of rabbit bone-marrow-derived macrophages | Macrophage differentiation |
| **Capsazepine** | 10 μM | Sigma-Aldrich | 30 min before each 50 J/cm^2^ irradiation; retained during irradiation and for 6 h afterward | TRPV1 pathway inhibition |
| **BAPTA-AM** | 10 μM | MedChemExpress | 30 min before each irradiation; retained during irradiation and for 6 h afterward | Intracellular Ca^2+^ chelation |
| **N-acetylcysteine (NAC)** | 5 mM | Sigma-Aldrich | 1 h before each irradiation; retained during irradiation and for 6 h afterward | ROS scavenging/redox-pathway interrogation |
| **MK-2206** | 1 μM | Selleck Chemicals | 1 h before each irradiation; retained during irradiation and for 6 h afterward | Allosteric Akt inhibition |
| **Meloxicam** | 10 μM | Sigma-Aldrich | 1 h before each irradiation; retained during irradiation and for 6 h afterward | Cyclooxygenase-sensitive comparison; not a specific mitochondrial CCO inhibitor |
| **SOX9-targeting siRNA** | 50 nM | GenePharma, Shanghai, China | Lipofectamine RNAiMAX transfection 48 h before first irradiation; repeated on day 7 for 14-day experiments | SOX9 knockdown |
| **Non-targeting control siRNA** | 50 nM | GenePharma, Shanghai, China | Same schedule as siSOX9 | Negative transfection control |
| **Lipofectamine RNAiMAX** | According to manufacturer instructions | Invitrogen | Used for siRNA transfection | siRNA delivery |

For daily pharmacological-intervention experiments, the medium was replaced 6 h after irradiation. Knockdown efficiency was verified by RT-qPCR and immunoblotting. CCO, cytochrome-c oxidase.

**Table S7. Bronchoscopic scoring criteria for repaired tracheal segments.**

| **Parameter** | **Score 0** | **Score 1** | **Score 2** | **Score 3** | **Score 4** |
| --- | --- | --- | --- | --- | --- |
| **Airway patency** | Complete/near-complete obstruction; <25% lumen patency | Severely compromised; 25-49% lumen patency | Moderately compromised; 50-74% lumen patency | Mildly compromised; 75-94% lumen patency | Nearly complete/complete patency; ≥95% lumen patency |
| **Granulation formation** | No visible granulation tissue | Minimal focal granulation; <10% of repaired region | Mild granulation; 10-25% of repaired region | Moderate granulation; 26-50% of repaired region | Marked/exuberant granulation; >50% of repaired region or obstructive growth |
| **Stenosis** | No stenosis or <5% lumen narrowing | Mild stenosis; 5-25% lumen narrowing | Moderate stenosis; 26-50% lumen narrowing | Severe stenosis; 51-75% lumen narrowing | Critical stenosis; >75% lumen narrowing or near occlusion |
| **Dynamic airway collapse** | No collapse or <5% dynamic lumen narrowing | Mild collapse; 5-25% dynamic lumen narrowing | Moderate collapse; 26-50% dynamic lumen narrowing | Severe collapse; 51-75% dynamic lumen narrowing | Near-complete/complete collapse; >75% dynamic lumen narrowing |

Lumen patency, stenosis and collapse were estimated relative to the adjacent normal tracheal segment. Dynamic collapse was assessed during spontaneous respiration. A higher airway-patency score indicates better repair, whereas lower granulation, stenosis and collapse scores indicate better repair. Two blinded observers scored each recording independently, and the mean score was used for analysis.
